# A bioinformatic survey of RNA-binding proteins in *Plasmodium*

**DOI:** 10.1186/s12864-015-2092-1

**Published:** 2015-11-02

**Authors:** BP Niranjan Reddy, Sony Shrestha, Kevin J. Hart, Xiaoying Liang, Karen Kemirembe, Liwang Cui, Scott E. Lindner

**Affiliations:** Department of Entomology, Center for Malaria Research, Pennsylvania State University, 501 ASI Bldg, University Park, PA 16802 USA; Department of Biochemistry and Molecular Biology, Center for Malaria Research, Pennsylvania State University, W223 Millennium Science Complex, University Park, PA 16802 USA

**Keywords:** RNA-binding proteins (RBPs), Post transcriptional regulation (PTR), Pre-mRNA splicing, Ribosome biogenesis, mRNA processing, Stress granules, Malaria, *Plasmodium*

## Abstract

**Background:**

The malaria parasites in the genus *Plasmodium* have a very complicated life cycle involving an invertebrate vector and a vertebrate host. RNA-binding proteins (RBPs) are critical factors involved in every aspect of the development of these parasites. However, very few RBPs have been functionally characterized to date in the human parasite *Plasmodium falciparum*.

**Methods:**

Using different bioinformatic methods and tools we searched *P*. *falciparum* genome to list and annotate RBPs. A representative 3D models for each of the RBD domain identified in *P*. *falciparum* was created using I-TESSAR and SWISS-MODEL. Microarray and RNAseq data analysis pertaining PfRBPs was performed using MeV software. Finally, Cytoscape was used to create protein-protein interaction network for CITH-Dozi and Caf1-CCR4-Not complexes.

**Results:**

We report the identification of 189 putative RBP genes belonging to 13 different families in *Plasmodium*, which comprise 3.5 % of all annotated genes. Almost 90 % (169/189) of these genes belong to six prominent RBP classes, namely RNA recognition motifs, DEAD/H-box RNA helicases, K homology, Zinc finger, Puf and Alba gene families. Interestingly, almost all of the identified RNA-binding helicases and KH genes have cognate homologs in model species, suggesting their evolutionary conservation. Exploration of the existing *P. falciparum* blood-stage transcriptomes revealed that most RBPs have peak mRNA expression levels early during the intraerythrocytic development cycle, which taper off in later stages. Nearly 27 % of RBPs have elevated expression in gametocytes, while 47 and 24 % have elevated mRNA expression in ookinete and asexual stages. Comparative interactome analyses using human and *Plasmodium* protein-protein interaction datasets suggest extensive conservation of the PfCITH/PfDOZI and PfCaf1*-*CCR4-NOT complexes.

**Conclusions:**

The *Plasmodium* parasites possess a large number of putative RBPs belonging to most of RBP families identified so far, suggesting the presence of extensive post-transcriptional regulation in these parasites. Taken together, *in silico* identification of these putative RBPs provides a foundation for future functional studies aimed at defining a unique network of post-transcriptional regulation in *P. falciparum*.

**Electronic supplementary material:**

The online version of this article (doi:10.1186/s12864-015-2092-1) contains supplementary material, which is available to authorized users.

## Background

Malaria continues to be a major public health and socio-economic problem in developing countries, and in 2013, it still caused 584,000 deaths (http://www.who.int/malaria/publications/world_malaria_report_2014/en/). Multifaceted control efforts are directed towards reducing malaria transmission, including vector control, early diagnosis, and effective treatment. Recently, the introduction of artemisinin combination therapies (ACTs) to deal with continually evolving multidrug resistance is a cornerstone of malaria chemotherapy, but this too is faltering and is spreading at a faster pace than anticipated [1]. As parasites continue to develop resistance to existing antimalarial drugs, continued research on developing new antimalarials remains a high priority [2]. One such approach has used systems biology methods in this postgenomic era of *Plasmodium* to identify multiple novel pathways in the parasite as potential drug targets [3–5]. Information gleaned from comparative genomic analysis and functional studies has contributed to improving our understanding of the parasite’s biology and our ability to design new control measures, and understanding basic regulatory mechanisms that parasite has evolved may help to guide future decisions in selecting targets.

The *Plasmodium* life cycle includes multiple stages with drastically different morphologies in a mosquito vector and a vertebrate host. This sophisticated developmental program requires regulation of gene expression and protein synthesis [6, 7]. Even with the discovery of the AP2-domain specific transcriptional factors [8], the parasite genome is still relatively deficient in identifiable transcriptional regulators [6], implying that post-transcriptional regulation (PTR) is an important means of regulation of gene expression. Furthermore, comparative studies examining the parasite’s transcriptomes and proteomes revealed significant lags in protein abundance relative to mRNA abundance [9]. During intraerythrocytic development, the half-life of mRNAs is substantially extended at the schizont stage when compared with that at the ring stage [10]. Translational regulation plays particularly critical roles during parasite transmission, when the parasites must remain relatively quiescent for an extended period of time before transmission occurs [11]. In the specific stages (gametocytes and sporozoites) that are transmitted, many mRNAs that are needed for subsequent development are kept in a translationally repressed state. Premature expression of these mRNAs leads to considerable defects in development [12, 13]. Altogether, these studies underscore the importance of post-transcriptional control in the development of the malaria parasite.

From transcription to degradation, every step of mRNA metabolism is subject to extensive regulation. Through mRNA maturation, export, subcellular localization, stability, and degradation, RNAs are accompanied by RNA-binding proteins (RBPs) and are thus found as messenger ribonucleoproteins (mRNPs). RBPs also play crucial roles in processing of stable RNAs such as rRNA, tRNA, snRNA, and snoRNA [14]. The significance of RBPs in translational regulation is underscored by their abundance in diverse eukaryotes. For example, the yeast *Saccharomyces cerevisiae* encodes ~600 RBPs [15], whereas in humans the number of RBPs is considerably larger with at least 1000 genes containing the RNA recognition motif (RRM) alone [16]. To date, more than a dozen RNA-binding domains (RBDs) have been identified and the best-characterized domains include RRMs, RNA helicases, zinc-finger domains (C3H1 and C2H2), K Homology (KH), Pumilio and Fem-3 binding factor (Puf), and Acetylation Lowers Binding Affinity (Alba) families. While most of our understanding about RBPs and their functions comes from studies of model organisms, their importance in the development of *Plasmodium* has recently been more appreciated [7, 11, 12, 17–20]. Given the potential roles of RBPs in virtually every aspect of RNA metabolism and in every part of the life cycle of the malaria parasites, we performed a comprehensive *in silico* analysis of RBPs in the malaria parasite *P. falciparum*. Many recent studies have also found that some RNA-interacting proteins may not possess commonly known RBDs [14], however, in this study we have used commonly known RBDs for the searches to ensure only more robust predictions are made. Using a set of bioinformatic tools, we identified 189 putative RBPs in the malaria parasite genome that contain well-characterized RBDs and provide functional annotation based on homology, domain organization, and expression patterns.

## Results and discussion

Using a combination of search strategies, we identified a total of 189 putative RBPs in the *P. falciparum* genome including 72 with the RRM, 48 putative RNA helicases, 11 with the KH domain, 2 with the Puf domain, 6 with the Alba domain, 31 with zinc fingers (ZnFs), and 19 other minor families of RBPs (Additional file 1). Most of these putative RBPs in *Plasmodium* lack definitive functional annotations. For functional predictions, each of these RBPs was BLAST searched against the model species by considering the total query sequence coverage against the template and the degree of domain-architecture conservation. This analysis allowed functional predictions for 140 putative RBPs (Additional file 1). While 179 of genes are conserved both in *Plasmodium vivax* and *Plasmodium yoelii* with clearly identifiable orthologs, 9 of the genes are lost in either or both *P. vivax* or *P. yoelii* (Additional file 1).

### RNA-binding domains and RBPs in *Plasmodium*

#### RNA-Recognition Motif (RRM)

**Fig. 1:**
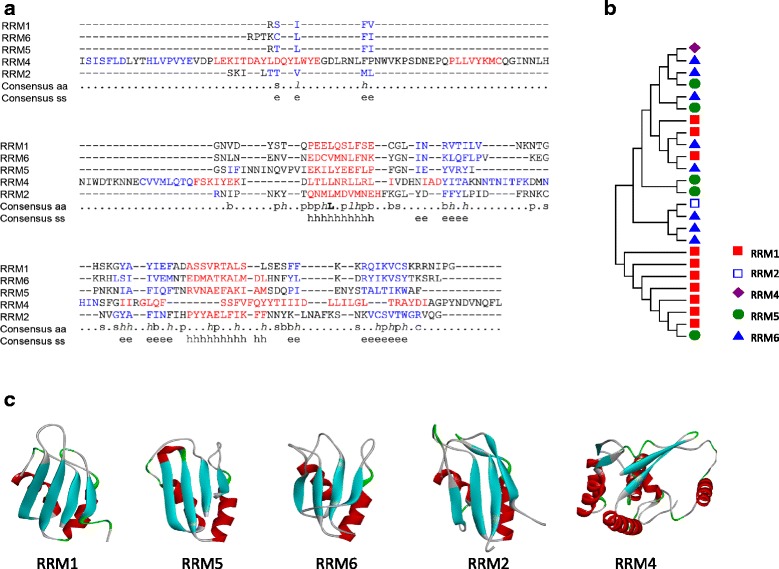
*P. falciparum* RRMs are divided into five RRM-families. **a** A multiple sequence alignment of 3D structures derived from representative members of each of the RRM families (RRM1-2, 4–6) found in *P. falciparum* is provided. RRM_4 is found to be highly diversified from typical RRM classes (RRM_1, RRM_5, RRM_6) followed by RRM_2. **b** Phylogenetic reconstruction of evolutionary relationship between RRM families from *P. falciparum*. Phylogenetic reconstruction of RRM families using representative domains from multiple PfRRMs failed to resolve the RRM families as expected, which may be due to relative number of RRMs used to represent each class (for example, RRM 2 and 4 have one domain each). **c** Representative 3D homology models for each of the RRM family were constructed using 3ucg, 3u1l, 2evz, 1p27 and 3zef PDB models as a reference to PF3D7_0923900, PF3D7_0515000, PF3D7_0606500, PF3D7_0623400, and PF3D7_0405400, respectively. It can clearly be seen that RRM4 (PfPrp8) is divergent from other members both at the primary sequence and structural level

The RRM is by far the most versatile and abundant RBD reported from bacteria to higher eukaryotes. The motif is about 70–90 amino acids in length and contains two consensus RNA-interacting motifs: RNP1 and RNP2. In the protein family database Pfam, RRMs are classified into ten different families based on profile similarities. We utilized representative sequences from individual RRM families as seeds to perform BLAST and hidden Markov model (HMM) searches in the *P. falciparum* genome to derive a final list of 120 RRM domains distributed in 72 proteins (Table 1). The number of RRM proteins in an organism appears to have increased through evolution, with higher-order species having more RRM proteins (Table 2). One exception is *Toxoplasma gondii*, a closely related species to *Plasmodium*, which encodes more than twice as many RRM proteins than *P. falciparum*. Compared with model organisms, *Plasmodium* species encode a similar number of RRM proteins as the yeast *S. cerevisiae*, which has a comparable genome size (Table 2). Five RRM families were found in *Plasmodium* genomes, whereas five other families (PF08777, PF10378, PF05172, PF10567 and PF14605) are completely absent. RRM_1 family is the most abundant with 55 members, followed by RRM_6 and _5 with 10 and 8 members, respectively. RRM_2 and _4 families only have one member (Table 1 and Fig. 1). Interestingly, RRM_2 family is supposedly specific to plants and fungi and is vastly expanded in plants (Table 2). The identification of the RRM_2 family member in *Plasmodium* suggests that this family in apicomplexans is likely derived from its red algae symbiont ancestor.

**Table 1:** List of different Pfam- and other profile families used to search RBPs from *P. falciparum* along with corresponding number of genes found in *P. falciparum*

RNA-binding domain (number of families)	Pfam id	Pfam id description	Number of corresponding genes in *P. falciparum*
RRM (8 families)	PF00076	RRM_1	55
	PF04059	RRM_2	1
	PF08777	RRM_3	0
	PF10598	RRM_4	1
	PF13893	RRM_5	8
	PF14259	RRM_6	10
	PF10378	RRM	0
	PF05172	Nup35_RRM	0
	PF10567	Nab6_mRNP_bdg	0
	PF14605	Nup53/35/40-type RNA recognition motif	0
RNA Helicases	PF00271	Helicase conserved C-terminal domain	63
	PF00270	DEAD helicase	51
	PF12513	Mitochondrial degradasome RNA helicase subunit C terminal	1
K Homology	PF00013	KH_1 (type I)	5
	PF07650	KH_2 (type II)	1
	PF13014	KH_3	0
	PF13083	KH_4	0
	PF13184	KH_5	0
	SSF54791	Eukaryotic type KH_domain I	9
	SSF54814	Prokaryotic type KH_domain II	2
Pumilio Homology Domain	PF00806	Pumilio	2
Alba	PF01918	Alba	6
C2H2 zinc finger	PF12171	zf-C2H2_jaz	2
	PF12756	zf-C2H2_2	1
	PF00641	zf-RanBP	1
	PF12874	zf-met	1
	PF12108	SF3a60_bindingd	1
	SM00355/SM00184	ZnF_C2H2/ Zinc finger, RING-type	4
	PS50157	ZINC_FINGER_C2H2_2	2
	PF00096	zf-C2H2	1
	PF06220	zf-U1	1
	PS50157	C2H2 type domain	1
	PF12171	zf-C2H2_jaz	2
C3H1	PF08772	NOB1_Zn_bind	1
	PF00642	zf-CCCH	2
	SM00356	Zinc finger	8
	PS50103	ZF_C3H1	9
PWI	PF01480	PWI domain	3
S-1 like	PF00575	S-1	4
SURP	PF01805	Surp module	2
G-patch	PF01585	G-patch	3
YTH	PF04146	YT521-B-like domain	2
PUA	SSF88697	PUA domain	5

**Table 2:** Comparative abundance of RRMs by Pfam class (including isoforms) across evolutionarily diverse species

Species name	PF00076 (RRM_1)	PF14259 (RRM_6)	PF13893 (RRM_5)	PF10598 (RRM_4)	PF04059 (RRM_2)	PF05172 (Nup_35)	PF10567 (Nab6)	PF14605 (Nup35_RRM_2)	Total
*Homo sapiens*	812	163	120	1	0	4	0	0	1100
*Arabidopsis thaliana*	505	105	51	3	15	2	0	7	688
*Drosophila melanogaster*	289	49	47	2	0	1	0	0	388
*Caenorhabditis elegans*	144	24	15	1	0	1	0	0	185
*Saccharomyces cerevisiae*	42	9	10	1	1	4	1	4	72
*Plasmodium falciparum*	55	10	8	1	1	0	0	0	75
*Plasmodium vivax*	56	10	8	1	1	0	0	0	76
*Plasmodium yoelii*	55	8	8	1	1	0	0	0	73
*Toxoplasma gondii*	137	19	20	2	5	0	0	0	183
*Cryptosporidium parvum*	30	4	7	1	0	0	0	0	42
*Trypanosoma cruzi*	51	5	4	1	0	0	0	1	62

Comparative inferences drawn from other species show that the presence of single and multiple RRMs in a protein is relatively common across different species [21]. Among the 72 RRM proteins in *P. falciparum*, 40 contain a single RRM, whereas 32 contain more than one RRM (Table 3 and Additional file 1). In addition, 16 of 72 RRM proteins have one or more of the 10 different types of other protein domains such as WWP repeating motif, Really Interesting New Gene (RING), C3H1 and C2H2 ZnF, G-patch, Suppressor-of-White-Apricot (SWAP), or poly(A) interacting domain (Table 3).

**Table 3:** The frequencies of occurrence of RRM in single, modular and multi-domain organization in *P. falciparum*

Single RRM (28 genes)		PF3D7_1367100, PF3D7_0923900, PF3D7_0503300, PF3D7_1002400, PF3D7_1224900, PF3D7_0515000, PF3D7_0319500, PF3D7_0415500, PF3D7_0615700
		PF3D7_0815600, PF3D7_0933000, PF3D7_1024200, PF3D7_1207500, PF3D7_1320900, PF3D7_1406000, PF3D7_1131000, PF3D7_1360100, PF3D7_0812500, PF3D7_0623400, PF3D7_1310700, PF3D7_1317300, PF3D7_1110400, PF3D7_1330800, PF3D7_0416000, PF3D7_0205700, PF3D7_1445600, PF3D7_1139100, PF3D7_1126800
Two RRM (21)		PF3D7_0414500, PF3D7_0920900, PF3D7_0935000, PF3D7_1306900, PF3D7_0629400, PF3D7_0517300, PF3D7_1004400, PF3D7_1119800, PF3D7_1006800, PF3D7_1022400, PF3D7_0916700, PF3D7_1420000, PF3D7_1020000, PF3D7_0728900, PF3D7_0606100, PF3D7_1107100, PF3D7_1405900, PF3D7_0723900, PF3D7_0929200, PF3D7_1022000, PF3D7_1326300
Three RRM (4)		PF3D7_1468800, PF3D7_1360900, PF3D7_1321700, PF3D7_1405900
Four RRM (2)		PF3D7_0606500, PF3D7_0716000
Five RRM (1)		PF3D7_1217200
RRM + ZnF (2)		PF3D7_1248200, Pf3D7_1244400
Znf + RRM + Znf (3)		PF3D7_1119300, PF3D7_0603100, PF3D7_1353400
RRM + SWAP + RPR (1)		PF3D7_1402700
RRM + WW + RRM (2)		PF3D7_1236100, PF3D7_0823200
Two RRM + WW + RRM (2)		PF3D7_1409800, PF3D7_1359400
Four RRM+ Poly(A) (1)		PF3D7_1224300
RRM + G patch (1)		PF3D7_1454000
RRM + RING finger (1)		PF3D7_1235300, PF3D7_1132100
Prp8 Multidomain (single RRM) (1)		PF3D7_0405400
RRM + WD40 (1)		PF3D7_0405400
RRM + PWI (1)		PF3D7_0610200

Blue , pink  and green  boxes are used to denote transmembrane, low complexity, and coiled-coil regions respectively

The average length of the RRM in *P. falciparum* is 75 aa (range 65–188 aa) (Additional file 2), which is similar to what has been reported in other species. Comparison of the different RRM families in *Plasmodium* found that the RRM_4 member Prp8 splicing factor is evolutionarily divergent from the other four families (Fig. 1a). Divergence of RRM_2 and RRM_4 family members from the other three major families is particularly noticeable in the RNA-binding motifs RNP1 and 2 (Fig. 1a). Phylogenetic analysis using only RRM-domain sequences of representatives from RRM_1-6 families failed to resolve evolutionary relationships as expected. For example, all RRM_1, 5 or 6 did not form monophyletic clades (Fig. 1b). Nonetheless, modeling of representative members of the five RRM families showed that the predicted structures conform to the typical organization of RRM and contains four anti-parallel beta strands and two alpha helices arranged as β_1_α_1_β_2_β_3_α_2_β_4_ (canonical RRM domain and RNP motifs are illustrated in Additional files 2 and 3) while showing sufficient diversity in overall 3D structures (Fig. 1c). For example, the RRM_4 family’s (Prp8) predicted 3D structure is highly diversified from the rest of the families.

Phylogeny-based orthology prediction identified one-to-one orthologs from *P. vivax* and *P. yoelii* except in two instances (PF3D7_1119800, PF3D7_1131000) where they were lost in *P. yoelii*. Both genes possess an SR domain and are predicted to participate in pre-mRNA splicing and export (Additional file 1). No recent duplications and species-specific expansion of RRM family genes were identified in a particular *Plasmodium* species (deficiency in paralogs), suggesting evolutionary constraints on independent evolution of the RRM gene family.

Phylogenetic analysis also identified four CUG-BP Elav-like (CELF) proteins and four potential poly(A)-binding proteins (PABPs) in *Plasmodium*. All CELF proteins have a similar multidomain organization with RRM domains flanking a variable WW domain, and they might have resulted from two gene duplication events (Table 3). PfCELF1 has recently been found to be a nuclear protein and participate in splicing [22]. Comparative bioinformatic analysis with human, *Drosophila* and *Arabidopsis* homologs classified the four *Plasmodium* PABPs into one nuclear and three cytoplasmic PABPs (Additional file 4). One cytoplasmic PABP (PfPABP1c) is evolutionarily conserved while the other three might have specifically acquired by *Plasmodium* species.

Because most of the *Plasmodium* RRM genes have not been characterized, we performed a variety of predictions of their functions. Thirty *P. falciparum* RRM proteins are predicted to participate in pre-mRNA splicing (13 genes), alternative splicing (10), transport (1), ribosome biogenesis (1), RNA degradation (1), translation (2), and post-transcriptional regulation (2). There are 25 other genes with different cellular functions while 17 genes are *Plasmodium*-specific with unknown functions (17) (Additional file 1). Functional analysis is needed to verify these predictions.

#### RNA helicases

Helicases are ubiquitous in nature and are considered to have evolved from near the very root of the evolutionary tree. Typically, helicases function in the separation of double-stranded RNA, DNA, and RNA/DNA structures in an energy-dependent manner [23]. Based on sequence similarities and domain conservation, helicases are classified into five superfamilies; superfamily II (SFII) is the most studied and most widely distributed in eukaryotes. Major components of SFII are DExD/H (Asp-Glu-x-Asp/His) helicase family members that primarily function in RNA metabolism including chaperoning snRNAs that participate in pre-mRNA splicing [24].

BLAST and HMM searches of the *P. falciparum* genome using three Pfam helicase families, PF00270 (DEAD/DEAH box helicases), PF00271, and PF12513, retrieved 51, 63 and 1 putative helicases (Table 1), respectively, similar to the number of helicases found in a previous study [25]. We further combined all three sets to derive a final set of 63 putative helicases in *Plasmodium*. Helicase members identified using PF00270 and PF12513 were all included in the set identified by using PF00271 as the seed. PF12513 is highly conserved from bacteria to eukaryotes and has one gene on average in each species, suggesting an early origin of this family. A previous text-based search of the *P. falciparum* genome retrieved 60 helicases, 22 of which with DEAD helicase family signatures [25]. With the lack of definitive features to bioinformatically classify helicases as DNA- and/or RNA-binding, it is generally considered that the DExD family preferentially binds RNA [26–28]. To circumvent difficulty in classifying RNA helicases, we performed a BLASTp search against five model species and trypanosomes with all putative helicases in order to predict their functions. This allowed us to retain 48 helicases as RNA helicases either due to the presence of an RNA-binding ortholog in other species or confirmation of binding to RNA in *P. falciparum*. Further mapping of the conserved motifs and domains classified 39 of them as DExD helicases (Additional file 5), which make up 80 % of total helicases in *P. falciparum*. Comparative genomic analysis showed that higher-order species have larger repertoires of helicases compared to lower strata, suggestive of lineage-specific evolution of the gene family. However, species in similar strata have comparable level of helicases; for example, *Plasmodium* spp. and *Toxoplasma* spp. have 60 and 73 helicases respectively (Table 4).

**Table 4:** A comparative table of helicases from different Phyla

Species name	All hits including isoforms	Unique sequences	Taxa ID
*Homo sapiens*	385	183	9606
*Arabidopsis thaliana*	239	172	3702
*Drosophila melanogaster*	226	96	7227
*Caenorhabditis elegans*	105	86	6239
*Saccharomyces cerevisiae*	206	74	4932
*Toxoplasma gondii*	73	73	508771
*Cryptosporidium parvum Iowa*	21	21	414452
*Plasmodium falciparum*	60	60	36329

Of the 48 RNA helicases, 28 contain a single helicase domain, whereas the remaining 20 contain additional domains such as helicase associated domain (HA2), oligonucleotide/oligosaccharide binding fold (OBNTP/OB fold), SPRY, Suv3, C2HC, S-1 and DSH C-terminal domain (DSHCT) (Table 5). Similar to the conservation of the RRM superfamily in *Plasmodium* spp., a search of the *P. vivax* and *P. yoelii* genomes with all putative helicases detected a 1:1 ortholog match in these species. Furthermore, each *Plasmodium* species has 30 and 9 DExD and DExH helicases, respectively, which is comparable to the numbers found in humans (36, 14) and *S. cerevisiae* (27, 7) [26]. This particular aspect, in conjunction with evolutionary inferences, highlights the conservation of these helicases across the species boundaries. This observation is further substantiated by the phylogenetic relationship among the helicases in *P. falciparum*. All the tree nodes have been consistently supported with high bootstrap values suggesting early origin of the helicases, which is also suggestive of evolutionarily conserved functions (Additional file 6).

**Table 5:** The frequencies of occurrence of RNA helicases in single, modular and multi-domain organization in *P. falciparum*

Name of the domain architecture	Domain architecture	Gene IDs
Helicase		PF3D7_0521700, PF3D7_0218400, PF3D7_1307300, PF3D7_1332700, PF3D7_0827000, PF3D7_1251500, PF3D7_0422700, PF3D7_1021500, PF3D7_1445900, PF3D7_0504200, PF3D7_0903400, PF3D7_1031500, PF3D7_1241800, PF3D7_0320800, PF3D7_0807100, PF3D7_0810600, PF3D7_1459000, PF3D7_1468700, PF3D7_0321600, PF3D7_0209800, PF3D7_0508700, PF3D7_0518500, PF3D7_0703500, PF3D7_0405000, PF3D7_1202000, PF3D7_0411400, PF3D7_0103600, PF3D7_1445200
HelicaseC + Suv3		PF3D7_0623700
Helicase + DUF4217		PF3D7_0721300, PF3D7_1419100, PF3D7_1418900, PF3D7_0630900
Helicase + ZnF		PF3D7_0527900, PF3D7_0909900, PF3D7_1313400
Helicase + UPF_Zn		PF3D7_1005500
Helicase + Sec63		PF3D7_1439100, PF3D7_0422500
Helicase + HA2 + S1		PF3D7_1030100
Helicase + HA2 + OB fold		PF3D7_1364300, PF3D7_1231600, PF3D7_0917600, PF3D7_0821300
Helicase + ZnF + DSHCT		PF3D7_0909900
Helicase + rRNA proc-arch + DSHCT		PF3D7_0602100
Helicase + HA2		PF3D7_0310500, PF3D7_1302700

Blue , pink  and green  boxes are used to denote transmembrane, low complexity, and coiled-coil regions, respectively

**Fig. 2:**
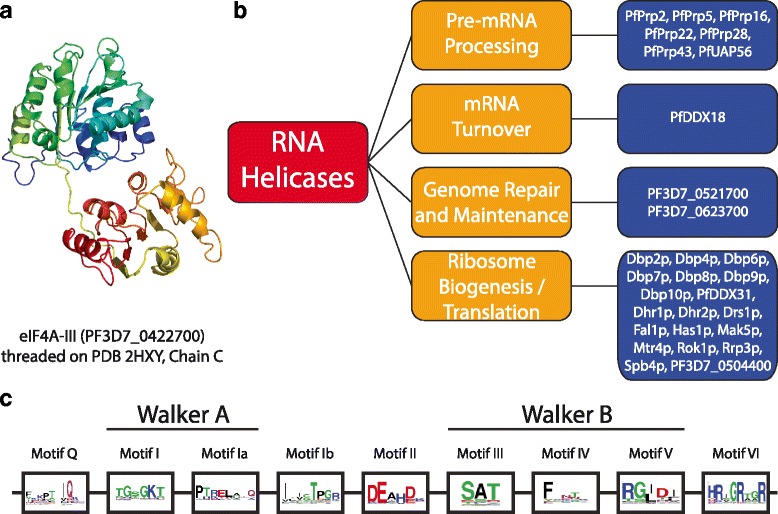
*P. falciparum* RNA-helicases retain the canonical conserved sequence motifs. **a** A representative 3D model of RNA helicase was constructed using PF3D7_0422700 (eukaryotic initiation factor) as a query and ATP-dependent RNA helicase DDX48 (PDB ID: 2hyi) as a template. **b** A categorization of putative functional roles of RNA helicases in *P. falciparum*. **c** A representation of the canonical, conserved catalytic RNA helicase domain is provided. Each functional unit of the helicase domain is divided into two functional units, Walker A and Walker B, which are further categorized into eight highly conserved sequence motifs named I, Ia, Ib and from II to VI. Walker A consists of an ATPase functional portion while Walker B has roles in ATP hydrolysis and nucleic acids unwinding [24]. The relative conservation of each of the conserved motifs in 42 PfRNA-helicases has been summarized in sequence logs. It can be seen that DExD/H at motif II is highly conserved suggestive of most of the RNA-helicases have this domain

To further illustrate the conservation of sequence motifs in RNA helicases in *Plasmodium*, a representative 3D model of RNA helicases was constructed using PF3D7_0422700 (eukaryotic initiation factor) as a query and ATP-dependent RNA helicase DDX48 (PDB ID: 2hyi) as a template (Fig. 2). All helicases have an evolutionarily conserved core structure made of two RecA-like, tandemly linked domains [29]. These domains possess all conserved residues required for nucleic acid binding (NAB), ATP binding and ATPase activities. At the sequence level, helicases are divided into two domains (Walker A and Walker B) with nine conserved motifs, Q, I, Ia, Ib and from II to VI [30]. Alignment of all 48 helicases and mapping the motif-specific sequence logos onto the 3D structure further confirmed the conservation in sequences and predicted structure (Fig. 2 and Additional file 5). Unlike RRMs, helicases are also highly conserved in their primary structure.

With regard to the functions of RNA helicases, generally DEAH helicases are involved in pre-mRNA processing, while DEAD helicases participate in ribosome biogenesis [26]. In *P. falciparum*, PF3D7_1364300, PF3D7_1231600, PF3D7_0917600 and PF3D7_1030100 all have a conserved DEAH domain and are classified as Prp (pre-mRNA processing) proteins. Similarly, almost all of the proteins classified under ribosome biogenesis (Fig. 2 and Additional file 6) have a conserved DEAD domain, indicative of evolutionary conservation of the protein synthesis apparatus. However, numerous exceptions to these rules have been observed, so these classifications should be experimentally confirmed and manually curated.

We performed a gene enrichment analysis using information on assigned biological processes as well as molecular functional information available from UniProt (http://www.uniprot.org/). From this analysis, 36 and 10 genes were classified as RNA-binding and mRNA processing, respectively, leaving the rest of the members unassigned. However, we could manually assign functions to 70 % of the RNA helicases from *P. falciparum* to ribosome biogenesis and related (17 genes), pre-mRNA processing (9), RNA degradation (3), mRNA turnover (1), genome repair and maintenance (2), and post-transcriptional regulation (2). Further corroborating the fact that helicases mainly take part in ribosome biogenesis, 30 of the 39 DExD/H helicases have a DExD domain (ribosome biogenesis), while 9 have a DExH domain (Additional file 5). Whereas 10 genes have homologs in model species without known functions, two genes (PF3D7_0103600 and PF3D7_1313400) appeared to be specific for the *Plasmodium* group. Though helicases are potential targets for drug design [31], very few of them have been characterized in *P. falciparum* [32, 33]. One such helicase (DOZI, a homolog of human DDX6 and yeast Dhh1) is essential to the development of the zygote in infected mosquitoes, and traffics a substantial portion of the mRNA pool to storage granules [12, 34, 35]. It would be interesting to see if *Plasmodium* specific helicases perform unique functions.

#### KH domain

The KH domain was first identified in the human heterogeneous nuclear ribonucleoprotein K (hnRNP) or pre-mRNA-binding protein K almost two decades ago [36]. The functional domain is about 70 aa in size, which primarily binds RNA [36–38]. KH domain proteins have a diverse regulatory portfolio, which includes transcription and translational regulation, RNA metabolism, and chromatin remodeling [37, 38].

**Fig. 3:**
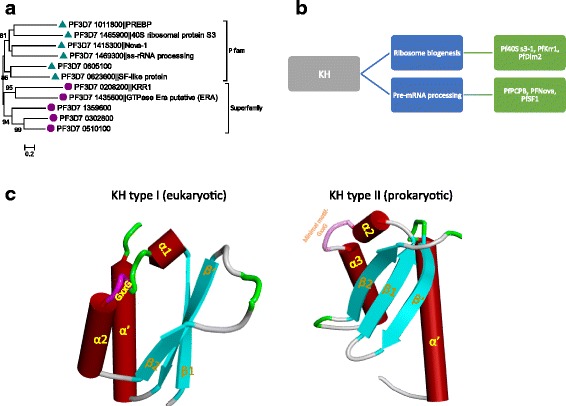
PfKHs are divided into two gene families based on their evolutionary origin and sequence conservation. **a** A phylogeny showing two monophyletic clades created from Pfam- and Superfamily-based retrievals. **b** Categorization of functional roles by KH domain genes in *P. falciparum* is provided. **c** A representative 3D model was constructed for type-I & type-II KH domain using PF3D7_1415300 and PF3D7_1465900 as queries using 2anr and 4d61, respectively. Typical secondary structure of type-I (β1α1α2β2 β’α’) & type-II KH domain (α’β’β1α1α2β2) are marked onto the model

BLAST and HMM searches of the *P. falciparum* genome using two different search criteria with Pfam families (PF00013, PF07650, PF13014, PF13083, and PF13184) and superfamilies (SSF54791, SSF54814) identified 19 KH domains in 11 genes (Table 1). Only two Pfam families (PF00013 and PF07650) identified 5 and 1 KH genes respectively, whereas searches using two superfamilies revealed the presence of additional five genes with KH domains. Phylogenetic analysis of KH domain genes found that the five genes identified using the two-superfamily sequences formed a monophyletic group (Fig. 3a), composed of members with predictable functions (Fig. 3b). Based on evolutionary origin and secondary structures, KH domain has been classified into two families—Type-I and Type-II [39]. Type-I mainly occurs in eukaryotes and can form modular structures, while type-II is of prokaryotic origin and mostly occurs alone [39]. Analyzing domain structure of *Plasmodium* KH domain proteins revealed 9 and 2 (PF3D7_1465900, PF3D7_1435800) type-1 and type-II members, respectively. The 3D homology models constructed using a type-I (PF3D7_1415300) and type-II (PF3D7_1465900) KH domain illustrate such differences in the two domain types (Fig. 3c). Conservation of these two prokaryotic genes that potentially function in ribosome biogenesis [40] suggests an early origin of the translational machinery. Two genes, PF3D7_0623600 and PF3D7_1435800 are found to occur with other domains (C2HC, MMR_HSR1 and Pduv_EutP) (Additional file 1).

Functional annotation through BLASTp search showed seven of the eleven KH domain genes have well-defined homologs in model species, allowing better prediction of their potential roles. Two KH domain genes are predicted to function in mRNA processing, three in ribosome biogenesis, one each in poly(A)- (PF3D7_1415300) and poly(rC)-binding (PF3D7_0605100), and in splicing (Fig. 3b). Interestingly, a recent study of a KH domain gene PF3D7_1011800 indicated it as a novel specific transcription factor [41]. This may be possible since some of the KH domains are found to interact with both RNA and ssDNA [38]. Similar to other RBPs, all the KH domain genes have orthologs in *P. vivax* and *P. yoelii*. We failed to detect homologs for four KH domain genes except in *Plasmodium* species, implying genus-specific evolution of KH proteins in malaria parasites.

#### Puf domains

Puf is named after the two founding members from *Pu*milio in *Drosophila* protein and *F*BF (fem-3 binding factor) in *Caenorhabditis elegans*. They represent an evolutionarily conserved class of translational repressors from a wide range of eukaryotic species, and are known to have diverse functions such as sexual differentiation and development, stem cell maintenance and neurogenesis [42, 43]. The Puf domain typically consists of eight homologous repeat units, each consisting of about 36 amino acids. Puf domains form a modular structure that can interact with eight ribonucleotides, with each repeat recognizing a single base. Two Puf proteins, Puf1 and Puf2 have been identified in all sequenced *Plasmodium* species (Puf domain-only alignment of PfPuf1, 2 is shown in Additional file 7) [7]. Homology modeling of the two Puf domains in *P. falciparum* showed a modular structure consistent with the typical Puf domain structure (Additional file 7). Puf1 and Puf2 have been characterized to regulate sexual development and transition from the mosquito vector to vertebrate hosts [11, 44]. Genetic deletion of Puf2 in *P. berghei* and *P. yoelii* leads to severe defects in sporozoite morphology and transmissibility, misregulation of mRNA transcript abundances, and in some cases affects male/female gametocyte ratios [12, 19, 45]. Over expression and knockdown of PfPuf2 expression in *P. falciparum* showed repression and elevation of gametocytogenesis, respectively [46]. A study by Miao et al. show that PfPuf2 regulates translationally repressed transcripts by interacting with Puf-binding elements (PBEs) located in both 3′- and 5′- untranslated regions [18]. For the first time, that study underscores the importance of 5′ UTRs in post-transcriptional regulation by PUF proteins, which now prompts investigations into additional regulation by PfPufs.

#### Alba

**Fig. 4:**
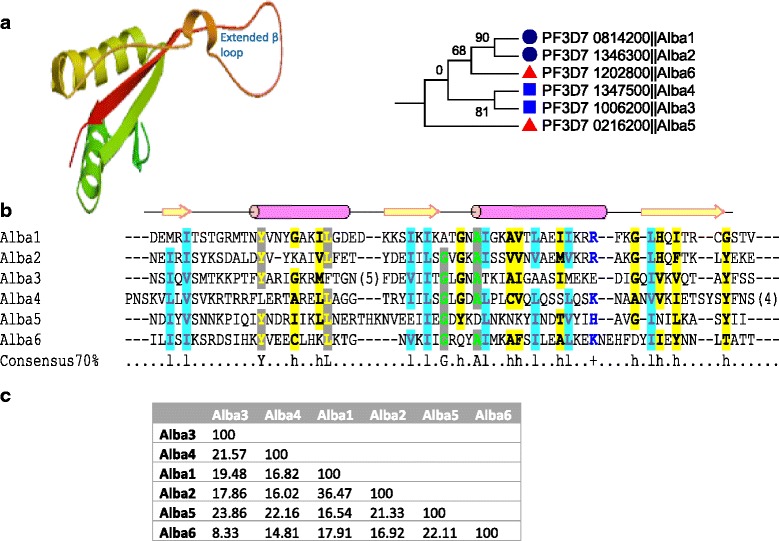
A comparison of identifiable ALBA proteins in *P. falciparum*. **a** A representative 3D model of an Alba domain is constructed using PF3D7_1346300 as a query and 2h9u as a template, and phylogenetic reconstruction of PfAlbas showing Alba1, 2 and Alba3, 4 are monophyletic groups. **b** A multiple sequence alignment of the Alba domain sequences from PfAlba1-6. Illustrated are the predicted secondary structural elements (arrow = alpha helix, block = beta strand) and conserved residues highlighted at 70 % consensus putatively interact with nucleic acids. Key for color-coded and highlighted amino acids letters are: negative DE; aliphatic ILV; positive MKR; tiny AGS; aromatic FHWY; charged DEHKR; small ACOGNPSTV; polar CDEHKNQRST; big EFIKLMQRWY; hydrophobic ACFGHIKLMRTVWY. The same color code is applied to rest of the alignments used in this manuscript. **c** A matrix of the percent identities for pairwise comparisons of PfAlbas 1–6 is provided

The Alba domain, formerly known as Sso10b, was first identified and characterized from a hyperthermophilic archaeon [47]. Recent studies confirmed its presence in all domains of life. Previous studies have characterized four Alba proteins (Alba1-4) in *Plasmodium*, which showed functional similarities to the canonical forms identified in *Sulfolobus* spp. [20, 48]. Using PF01918 and profile searches against *P. falciparum* genome in HAMMER, we identified two new members (PfAlba5: PF3D7_0216200 and PfAlba6: PF3D7_1202800) (Fig. 4a). PfAlba6 is highly diverged from rest of the group with only limited sequence identities with other *Plasmodium* Alba proteins (Fig. 4b and c). Phylogenetic reconstruction showed PfAlba1-2 and 3–4 formed two separate monophyletic clades leaving newly identified Albas as singletons (Fig. 4a). Interestingly, out of these four, three genes have undefined homologs in *Arabidopsis* suggesting their evolutionary conservation. BLAST searches with lower E-value (10) failed to identify homologs outside Apicomplexa suggesting possible lineage-specific evolution of PfAlba5 and 6. It is therefore interesting to see the functions of these putatively novel genes in *Plasmodium* species. To further map the conserved nucleic acid binding interface of PfAlbas, domain-only specific sequences with the conserved residues at 70 % of consensus level were extracted and mapped, which illuminated that the amino acid positions putatively interacting with DNA/RNA are also conserved in PfAlba5, 6 (Fig. 4b). A 3D model of PfAlba2 (PF3D7_1346300) with the archaea-specific DNA-binding protein (PDB ID: 2h9u) as the template showed 27 % identity through 77 % of query coverage (Fig. 4a). Typically Alba domains form a homodimer of two 10 kDa subunits. The predicted PfAlba2 model showed the conserved feature of an extended β sheet hairpin loop [47]. PfAlba proteins exist as a single domain as well as in association with other functional domains such as RGG box—a RNA-binding motif in PfAlba1 and 2 [20]. Alba proteins are conserved with corresponding orthologs in other *Plasmodium* species (Additional file 1).

The Alba domain has been implicated in transcriptional and translational regulation through its ability to bind both DNA and RNA, and due to its association with Sir2 [49, 50]. Functional annotation of PfAlbas is not possible based on homology searches of genomes of model organisms. Whereas homologs of Alba1-3 were found in *Arabidopsis* with unknown functions, we did not identify homologs of Alba4-6 in model organisms even after relaxing the search parameters, suggesting a lineage-specific evolution. Similar to the canonical Alba proteins, PfAlba1-4 were reported to bind both DNA and RNA [20, 48]. Several Alba proteins from Apicomplexa (including *Plasmodium*) were reported to be involved in diverse cellular functions such as binding and regulating their own transcripts, regulating transcription through condensation of chromatin, and post-transcriptional regulation of mRNAs involved in development [49–51]. PfAlba1 is essential for asexual erythrocytic development and binds to ~30 % of the trophozoite transcriptome, regulating the timing of the translation [52]. Yeast two-hybrid data revealed interactions between PfAlba3 and 4. Similar observations were made for *Toxoplasma* TgAlba2 and TgAlba1, where the former depends on the latter for expression [51]. In *P. berghei*, PbAlba1-4 were associated with the DOZI and CITH translational repression complexes, confirming their roles in *Plasmodium* RNA biology [13].

#### Zinc finger domain

Zinc Finger (ZnF) domains are small protein domains present in all forms of life and are one of the most studied domains in transcription factors. The functional versatility of the ZnF-containing proteins arises from the modular structure of ZnFs, which can be found in multiple copies and in different forms. At least 46 different types of ZnFs have been identified in mammalian transcriptomes [52]. ZnFs are classified into various groups based on structural similarities, including the number of zinc ligands they bind, and the arrangement and the number of cysteine (C) and histidine (H) residues surrounding one or more zinc atoms [53]. ZnFs can bind DNA, RNA, or protein, and the distance between two ZnF domains on a protein critically influences these interactions. The most characterized forms of RNA-binding ZnF forms are C2H2 and C3H1, which fold to create RNA-binding surfaces composed of α-helices and aromatic side chains [54].

Using various Pfam and other profile families as seed sequences (Table 1), we retrieved a total of 31 putative RNA-binding ZnF proteins. Of which, 20 and 11 genes belong to the C3H1 and C2H2 forms, respectively. Both C3H1 and C2H2 ZnFs coexist with other protein domains such as the RRM, RING, YTH, and PWI domains (C3H1) and the CactinC and RANB2 domains (C2H2) (Additional file 1). Based on homology searches, functional annotation was possible for eight of the eleven C2H2 genes; five genes may be involved in splicing and two in ribosome biogenesis. For 18 of the 20 C3H1 genes, specific functions could not be ascertained due to lack of orthologs in model species (Additional file 1).

#### Other potential RBDs

In addition to the major RBDs described above, we identified several minor RBP families including proteins containing the pseudouridine synthase and archaeosine transglycosylase (PUA) domain, YT521-B homology, S-1 motif, SWAP (Suppressor-of-White-APricot domains), PWI, and G-patch motif. All these minor domains have predicted orthologs in *P. vivax* and *P. yoelii* genomes.

The PUA is a compact 67–94 aa motif frequently found in RNA modification enzymes and nucleoproteins [55]. The motif is also commonly found in other proteins that have functional roles in translation and ribosome biogenesis [55]. Our analysis revealed five PUA containing genes (Additional file 1). Functional annotation of these genes indicates that they may have potential roles in tRNA and rRNA post-transcriptional modifications and maturation, RNA methylation, and translation initiation. In *Plasmodium*, the PUA domain is found to coexist with the S-adenosyl methionine domain (important for methylation functions) and the DKCLD domain (a TruB_N/PUA domain variant associated N-terminal domain of Dyskerin-like proteins).

The YTH (YT521-B homology-a part of PUA domain superfamily) constitutes a new class of RBP in eukaryotes [56], which was first identified and characterized in the YT521-B protein [57]. The domain is typically 100–150 aa in length, and is rich in aromatic residues that are reminiscent of RRM and PUA domains [56]. The domain is found to have functions in alternative splicing and the prevention of untimely meiosis in yeast through the degradation of meiosis-specific transcripts during vegetative growth [58]. Two genes were identified in the *P. falciparum* genome (PF3D7_0309800 and PF3D7_1419900) that encode this domain and other putative RBDs such as the C3H1 ZnF (Additional file 1). *In silico* functional annotation suggests that the YTH domain may participate in modulating alternative splicing, mRNA cleavage and polyadenylation in *P. falciparum*.

The S1 motif was first identified in *E. coli* ribosomal S1 protein and exhibits an evolutionarily conserved nucleic acid binding OB (oligonucleotide/oligosaccharide binding) structural fold [59]. The S1 motif in *P. falciparum* was found to co-exist with other RBDs such as KH and RNA helicase domains. These proteins may be involved in pre-mRNA processing, ribosome biogenesis and translation in *Plasmodium* (Additional file 1).

The SWAP domain was first identified in *Drosophila* splicing regulators. Pfam searches of the *P. falciparum* genome revealed the presence of two genes with the SWAP domains, namely PF3D71474500 (splicing factor 3A) and PF3D7_1402700 (pre-mRNA splicing factor). While PF3D7_1474500 has two SWAP domains, the PF3D7_1402700 has one SWAP domain with one RRM (Additional file 1).

The PWI domain is an another RNA-binding domain first reported in splicing factors [60, 61]. Of the three PWI-containing genes in *P. falciparum*, one (PF3D7_0610200) also has an N-terminal RRM domain. PWI genes may play roles in splicing and alternative splicing in *Plasmodium* (Additional file 1).

The glycine-rich nucleic acid binding domain called G-patch was first described by Aravind and Koonin [62]. We identified three G-patch genes (PF3D7_1454000, PF3D7_1110300, and PF3D7_0531400) in *P. falciparum* genome. Only PF3D7_1454000 is associated with an RRM (Additional file 1).

### Functional roles of *Plasmodium* RBPs

RBPs are at the center of RNA metabolism and involved in all aspects of RNA biology. Based mostly on homology with RBPs in model organisms with known functions, we manually annotated the predicted functions of some putative RBPs in *Plasmodium* and categorized them into various cellular processes.

#### RBPs in splicing

Splicing of precursor mRNAs is carried out by a specialized, massive ribonucleoprotein (RNP) complex termed the spliceosome, which is highly conserved in eukaryotes. The spliceosome consists of five small nuclear ribonucleoproteins (U1, U2, U4/U6, U5 snRNPs) and non-snRNPs such as serine/arginine-rich (SR) family proteins [63]. Although splicing in *Plasmodium* remains to be fully characterized [64], some conserved components of the splicing machinery have been identified [31, 48, 65–67], including five snRNAs [66, 68] and 28 RBPs with putative functions in pre-mRNA splicing (Table 6). Among them, 13 and 6 proteins belong to the RRM and RNA helicase families, respectively. All of the major spliceosome initiation factors—U2AF65, U2AF35, SF1, SF3b, *P*re-*R*NA *p*rocessing (Prp) 5, Prp28, SF3A3, SNRPC, ZRANB2, and Snu23 are encoded by the *Plasmodium* genome. In addition, proteins involved in the proofreading of the splicing and joining processes such as Prp16, Prp22, and Prp43 were also identified in the *Plasmodium* genome [69] (Additional file 1). Pfprp16 has been shown to bind RNA and hydrolyze ATP in the presence of helicase associated domain (HA2) [70].

**Table 6:** List of genes and their putative functions involved in splicing mechanism in *P. falciparum*

Gene name	Putative function	Common name
PF3D7_0515000	Pre-mRNA-splicing factor Cwc2	PfCwc2
PF3D7_1224900	Splicing factor 3B subunit 6 (SF3B6)	PfSF3B6
PF3D7_1420000	Splicing factor 3B subunit 4 (SF3B4)	PfSF3B4
PF3D7_0935000	U2 snRNP associated small nuclear ribonucleoprotein B	PfsnRPB2-B
PF3D7_1367100	U1 small nuclear ribonucleoprotein 70 kDa	PfU1snRNP
PF3D7_1306900	U1 snRNP assocaited small nuclear ribonucleoprotein A	PfsnRPBU1-A
PF3D7_1402700	U2 snRNP-associated SURP motif-containing protein	PfsnRPB2-2
PF3D7_1326300	Splicing factor homolog	PfSfx1
PF3D7_0716000	Splicing factor homolog	PfSfx2
PF3D7_1468800	Splicing factor U2AF large subunit B	PfU2AF3
PF3D7_1119300	Splicing factor U2AF small subunit B	PfU2AF4
PF3D7_1321700	Splicing factor, CC1 like	PfRBM39
PF3D7_0209800	Spliceosome RNA helicase DDX39B; alias UAP56	PfUAP56
PF3D7_0812700	U1 small nuclear ribonucleoprotein C (SNRPC)	PfSNRPC
PF3D7_0408300	Supraspliceosme complex component -alternative splicing	PfZRANB2
PF3D7_0209800	Spliceosome RNA helicase DDX39B; alias UAP56	Pf UAP56
PF3D7_0508700	Pre-mRNA-processing ATP-dependent RNA helicase Prp5	PfPrp5
PF3D7_0518500	ATP-dependent RNA helicase DDX23 (PRP28)	PfPrp28
PF3D7_1443800	Mdlc (midlife crisis) or Cwc24p in yeast	Pfmdlc
PF3D7_0623600	Splicing factor 1 (SF1)	PfSF1
PF3D7_1474500	Splicing factor 3A subunit 1 (PRP-21)	PfPrp21
PF3D7_0619900	REPO-1	PfPrp11
PF3D7_0924700	Splicing factor 3a, subunit 3, 60 kDa (SF3A3)	PfPrp9
PF3D7_0525000	Putative poly-adenylation factor	Ambiguous
PF3D7_1443800	mdlc (midlife crisis) or Cwc24p in yeast	Pfmdlc1p
PF3D7_1364300	Pre-mRNA-splicing factor ATP-dependent RNA helicase PRP16	PfPrp16
PF3D7_1030100	Pre-mRNA-splicing factor ATP-dependent RNA helicase PRP22	PfPrp22
PF3D7_0917600	Pre-mRNA-splicing factor ATP-dependent RNA helicase PRP43	PfPrp43
PF3D7_0606500	Polypyrimidine tract-binding protein 3	PfPTBP1
PF3D7_1409800	RNA binding protein Bruno, putative (HoBo) Bruno	PfCELF1
PF3D7_0823200	CUG-BP Elav-like family member 3	PfCELF2
PF3D7_1236100	CUGBP, Elav-like family member 2	PfCELF3
PF3D7_1022400	Pre-mRNA-splicing factor SF2	PfSF2
PF3D7_1454000	Splicing factor 45	PfSpf45
PF3D7_0517300	Splicing factor, arginine/serine-rich 1	PfRSrrm1
PF3D7_1004400	Serine/arginine-rich splicing factor 4	PfRSrrm2
PF3D7_1119800	Serine/arginine-rich splicing factor 1	PfRSrrm3
PF3D7_0503300	Serine/arginine-rich SC35-like splicing factor SCL28	PfRSrrm4
PF3D7_1006800	Gbp2p	PfRSrrm5
PF3D7_1002400.1	Transformer-2 protein homolog beta isoform 2 (TRA2B)	PfRSrrm6
PF3D7_1415300	Nova2 or BTR1	PfNova2
PF3D7_0309800	YT521	PfYT521

Alternative splicing creates multiple transcripts from a single gene, thus contributing to the diversity of the cellular proteome without a need for genomic expansion. While 95 % of multi-exon genes have more than one transcript isoform in humans, alternative splicing also occurs in *P. falciparum,* albeit to a much lesser extent [64, 71–73]. RNA-seq analyses of the *P. falciparum* transcriptomes found evidence for alternative splicing in about 300 genes [64, 71]. Through bioinformatic analysis, we identified 13 genes in *P. falciparum* with predicted roles in alternative splicing (Table 6). Most of these genes are from the SR (7 genes) and the CELF (4 genes) families. SR family proteins have RRM domain(s) and arginine-serine repeats. Two SR genes in *P. falciparum* (PfSrrm1 and PfRSrrm3) were shown to bind to RNA [68, 79], and PfSrrm1 was predicted to regulate alternative splicing [74]. PfSF2, a homolog of serine/arginine-rich splicing factor 1(AF1) or pre-mRNA-splicing factor SF2 (SF2) was predicted to function in alternative splicing in *P. falciparum* and affected parasite proliferation in erythrocytes [74]. The CELF/Bruno-like family RBPs regulate pre-mRNA splicing/alternative splicing in the nucleus, as well as mRNA deadenylation and translation in the cytoplasm [75–77]. Of the four *Plasmodium* CELF family genes, PfCELF1 was characterized to function in pre-mRNA processing [22]. The polypyrimidine tract binding proteins (PTBPs), a family of multiple RRM domain containing proteins, regulate alternative splicing by binding to the polypyrimidine regulatory tracts that exist in introns [78, 79]. While at least two PTBPs are found in the human genome, we only identified one PTBP-like protein, PfPTBP1, in the *P. falciparum* genome (Table 6).

#### RNA maturation, exon-exon junction complex formation and mRNA shuttling

RNA maturation in eukaryotes includes 5′ methyl capping and 3′ poly (A)-tailing of mRNAs. These processes are predicted to be conserved in malaria parasites. Among them, PF3D7_1419900 is a homolog of the 30 kDa subunit of human cleavage and polyadenylation specificity factor (CPSF), an RNA-binding endonuclease playing a role in 3′ processing of pre-mRNA [80]. Following complete maturation, export of mRNAs to the cytoplasm is achieved by a special mRNP complex termed the exon-exon junction complex (EJC) [81, 82]. It is comprised of a mixture of mRNA export factors—Aly/REF, TAP, Upf3b, UAP56 [67], and nonsense mediated mRNA surveillance (NMD) components—Y14 and Magoh. Our analysis identified all of the known homologs of both EJC and NMD complexes; however, their predicted functions have yet to be confirmed in *P. falciparum* except for PfUAP56 which was shown to harbor RNA binding and helicase activities that depend upon glycine 181, isoleucine 182 and arginine 206 [67].

#### RBPs in ribosome biogenesis and translation initiation

Ribosome biogenesis in eukaryotes involves the processing of rRNAs, assembly of the 40S and 60S subunit precursors in the nucleus, and export of the precursors to the cytoplasm. Most of the ribosomal proteins fall into various energy-consuming enzyme families including the ATP-dependent RNA helicases. Comparative genomic analyses using the yeast proteins involved in ribosome biogenesis identified 14 *P. falciparum* helicases with potential roles in this process (Table 7). Interestingly, all but one (Dbp9p) helicase homolog involved in ribosome biogenesis was identified in *Plasmodium*. These helicases are further divided into eight and nine helicases involved in small subunit and large subunit pre-processing, respectively. Similar to other RBP classes, all of these homologs remain to be experimentally characterized in *P. falciparum* (Table 7).

**Table 7:** A list of genes and their putative functions involved in ribosome biogenesis in *P. falciparum*

Gene ID	Putative function	Named in *P. falciparum*	Remarks
PF3D7_0218400	DDX47 (Rrp8p)	PfRrp8p	*18S rRNA processing, participates in cleavages at A_2_, and to a lesser extent, A_0_ and A_1_ sites
PF3D7_0721300	DDX31 (Dbp7p)	PfDbp7p	27S pre-ribosomal rRNA processing (60S ribosomal subunit biogenesis) [123]
PF3D7_1419100	DDX55 (Spb4p)	PfSpb4p	*5.8S/25S pre-ribosomal rRNA processing (60S ribosomal subunit biogenesis)
PF3D7_1418900	DDX10 (Dbp4p)	PfDbp4p	18S rRNA processing
PF3D7_1307300	DDX18 (Dbp6p)	PfDbp6p	*27S pre-rRNA processing (60S ribosomal subunit biogenesis)
PF3D7_1332700	DDX49 (Rrp3p)	PfRrp3p	*60S ribosomal subunit assembly-27S pre-rRNA processing
PF3D7_0827000	DBP10 (DBP10) or DDX54 isoform 1	PfDbp10p	*5.8S/25S rRNA processing
PF3D7_1251500	DDx27 (Drs1p)	PfDrs1p	*27S- > 25S rRNA conversion (60S ribosomal subunit biogenesis)
PF3D7_0422700	EIF4A3 (Fal1p)	PfFal1p	*18S rRNA processing, participates in cleavage at A_0,_ A_1_ and A_2_ sites
PF3D7_1021500	DDX52 (Rok1p)	PfRok1p	*18S rRNA processing, participates in cleavage at A_1_ and A_2_ sites
PF3D7_0527900	DDX41 (Mak5p)	PfMak5p	*60S ribosome subunit assembly
PF3D7_1302700	DHX37 (dhr1p)	PfDhr1p	*18S rRNA processing, participates in cleavage at A_0,_ A_1_ and A_2_ sites
PF3D7_1445900	DDX17 isoform 1 (Dbp2p)	PfDbp2p	*60S ribosomal subunit biogenesis
PF3D7_0602100	SKIV2L2 or Mtr4p	PfMtr4p	*5.8S rRNA processing
PF3D7_0630900	Has1p	PfHas1p	Maturation of 40S and 60S ribosomal subunits
PF3D7_0504400	DDX21	PfDdx21p	RNA processing and nucleolar localization
PF3D7_1217200	Mrd1p	PfMrd1p	Release of base-paired U3 snoRNA within the pre-ribosomal complex [124]
PF3D7_0409800	Rei1p	PfRei1p	It has functional redundancy with yeast proteins Reh1 in cytoplasmic 60S subunit maturation
PF3D7_1464400	Bud20p	PfBud20p	Helps in shuttling pre-ribosomal 60S complex to cytoplasm; U1-like Zn-finger-containing protein
PF3D7_1474500	Splicing factor 3a	PfSF3a	Splicing of rRNA genes
PF3D7_1465900	40S ribosomal protein S3-1	Pf40S s3-1p	Multifaceted functional roles; involves in translation, binding to DNA, and regulating transcription of specific set of genes
PF3D7_0208200	KRR1	PfKrr1p	Synthesis of 18S rRNA (SSU) processome component
PF3D7_1469300	Pno1p or Dim2p	PfDim2p	Shuttling of Dim1 rRNA from cytoplasm to nucleolus
PF3D7_1466700	NIP7 homolog	PfNip7p	60S ribosome subunit biogenesis protein NIP7 homolog isoform 1; nucleolar pre-rRNA processing
PF3D7_1417500	NAP57	PfNap57p	Pseudouridine synthase NAP57 or H/ACA ribonucleoprotein complex subunit 4 (5e-178), *H. sapiens*
PF3D7_0907600	SUI1 family protein	PfeIF	Eukaryotic translation initiation factor SUI1 family protein isoform 1 (formerly named as ligetin)
PF3D7_0529500	MCTS1	PfMcts1	May be initiation factor homolog
PF3D7_1450600	SAM dependent methyltrasferase	PfSam	RNA methylation
PF3D7_0418700	RNA-binding protein NOB1	PfNob1p	Biogenesis of 40S rRNA through cleavage of D-site in 20S rRNA

Entries marked with an asterisk (“*”) were retrieved from [122]

#### RBPs in genome repair and maintenance

Genome repair and maintenance are crucial for the integrity of the genome. Based on a homology search, we identified two RBPs from the *P. falciparum* genome that have putative functions in genome maintenance. Human DDX1 is reported to be activated by phosphorylation in response to double-stranded breaks in DNA. DDX1 has RNase activity towards single-stranded RNA as well as ADP-dependent RNA-DNA- and RNA-RNA-unwinding activities [83, 84]. The putative DDX1 homolog from *Plasmodium* (PF3D7_0521700) is highly conserved with 29 % identity at 93 % total gene coverage. Another gene, PF3D7_0623700 has a C-terminal domain resembling the yeast Suv3p protein, which is associated with mitochondrial genome stability [85, 86].

#### RBPs in RNA granules, degradation and translational regulation

RNA granules (stress granules, storage granules, P-bodies, P-granules) formed during stress and non-stress conditions provide a well-conserved means for a cell to regulate its gene expression. Although they all regulate RNA homeostasis in a cell, their compositions and functions are different. Moreover, the classification and functional assignment of these granules is fluid, as they are now thought to exist in a continuum and are only loosely defined by the presence/absence of various protein and RNA components [87]. Classically, stress granules form in response to different stressors, for example depletion of glucose. Stress granules typically contain translation initiation factors (eIF2, eIF3, eIF4G, eIF4A, eIF4B, and eIF4E) and PABPs [88]. Putative components of stress granules, the exosome, and processing bodies (P-bodies) found in the *P. falciparum* genome are listed in Table 8. It is important to note that few of these proteins have been experimentally validated to associate with granules in *Plasmodium*, and that experimental confirmation of this is certainly warranted. P-bodies are seen in the presence and absence of stress, and the composition of P-bodies is likely independent of the stressor. P-bodies differ from stress granules, as they contain proteins associated with mRNA degradation to decap and deadenylate transcripts. There are 13 core, canonical P-body proteins that include XRN1, HCCR4, DCP1, DCP2, and eIF4E, to name a few [89–91]. In *Plasmodium*, BLASTp alignments with *Plasmodium* proteins identified predicted orthologues of DCP, RCK1, LSM1-7, XRN1, and Rap55 (11 of the 13 core components) (Table 8). The predicted DCP1 and DCP2 proteins share homology with the DCP1 superfamily domain and the NUDIX domain, respectively, thus strengthening these assignments. In contrast, no DCPS ortholog was identified even with relaxed search parameters. RCK, which is also a decapping activator, has been identified in *Plasmodium*. These proteins that likely traffic to cytosolic granules are important to the development and transmission of the parasite. During development of eukaryotes, many mRNAs are stored in a translationally repressed state in storage granules like the P- granules in metazoan germ cells. Similarly, *P. berghei* gametocytes produce a P-granule-like storage granule, which contains the RNA helicase DOZI, the Sm-like factor CITH, PABPs, Bruno homolog, the Mushashi homolog, and four Alba proteins [13]. Moreover, the DOZI complex was found to associate with a substantial portion of the transcripts found in gametocytes [35]. The components of this RNA granule are highly conserved across *Plasmodium* species.

**Table 8:** The inferred contents of exosomes, P -bodies, and stress granules in *Plasmodium* species. The composition of RNA granules in *Plasmodium* was inferred by conducting BLASTp queries using the amino acid sequences of components of exosomes, P bodies, and stress granules from model organisms (*D. melanogaster*, *S. cerevisiae*, *C. elegans*) against known and predicted *Plasmodium* amino acid sequences. Other *Plasmodium* proteins that traffic to granules, but that cannot be definitively placed in a currently annotated granule type, are listed separately. Gene identifiers for these proteins for three commonly studied malaria species (*P. falciparum*, *P. vivax*, *P. yoelii*) were obtained from PlasmoDB.org

Exosome	*P. falciparum* Gene ID	*P. vivax* Gene ID	*P. yoelii* Gene ID
Csl4	PF3D7_0720000	PVX_096320	PY17X_0620200
Rrp4	PF3D7_0410400	PVX_000730	PY17X_1009400
Rrp40	PF3D7_1307000	PVX_122185	PY17X_1407200
Rrp41	PF3D7_1427800	PVX_085150	PY17X_1018300
Rrp42	PF3D7_1340100	PVX_082925	PY17X_1358900
Rrp45	PF3D7_1364500	PVX_115185	PY17X_1141800
Rrp6	PF3D7_1449700	PVX_118000	PY17X_1317200
Rrp44/Dis3	PF3D7_1359300	PVX_114935	PY17X_1137100
Mpp6 (Accessory)	PF3D7_0928900	PVX_099895	PY17X_0833000
RNaseII	PF3D7_0906000	PVX_098745	PY17X_0418100
P Bodies	*P. falciparum* Gene ID	*P. vivax* Gene ID	*P. yoelii* Gene ID
BRF1	PF3D7_1449300	PVX_118025	PY17X_1316800
NOT1	PF3D7_1103800	PVX_090876,	PY17X_0945600
		PVX_090878	
HCCR4-Like	PF3D7_0519500	PVX_080270	PY17X_1237700
CAF1	PF3D7_0811300	PVX_123205	PY17X_1428300
CNOT3	PF3D7_1006100	PVX_094500	PY17X_1207500
CNOT2	PF3D7_1128600	PVX_092050	PY17X_0921700
CNOT4	PF3D7_1235300	PVX_100715	PY17X_1452400
ABCA10	PF3D7_1434000	PVX_084835	PY17X_1012400
NOT9	PF3D7_0507600	PVX_097940	PY17X_1108300
NOTx	PF3D7_1417200	PVX_085590	PY17X_1027900
DCP1	PF3D7_1032100	PVX_111120	PY17X_0517000
DCP2	PF3D7_1308900	PVX_122275	PY17X_1409100
EIF3	PF3D7_0517700	PVX_080365	PY17X_1235900
eIF4E	PF3D7_0315100	PVX_095480	PY17X_0415700
eIF4G	PF3D7_1312900	PVX_122470	PY17X_1413100
eRF1	PF3D7_0212300	PVX_002915	PY17X_0309700
eRF3	PF3D7_1123400	PVX_091785	PY17X_0926900
LSM1	PF3D7_1124400	PVX_091835	PY17X_0925900
LSM2	PF3D7_0520300	PVX_080230	PY17X_1238500
LSM3	PF3D7_0819900	PVX_089370	PY17X_0711100
LSM4	PF3D7_1107000	PVX_091025	PY17X_0942400
LSM5	PF3D7_1443300	PVX_118325	PY17X_1311000
LSM6	PF3D7_1325000	PVX_116625	PY17X_1344900
LSM7	PF3D7_1209200	PVX_084490	PY17X_0610100
Pab1	PF3D7_1224300	PVX_123845	PY17X_1441700
Rpb4	PF3D7_1404000	PVX_086235	PY17X_1040500
Rbp7	PF3D7_1104700.1,	PVX_090915	PY17X_0944700
	PF3D7_1104700.2		
Sbp1	PF3D7_0501300	PVX_097583	
Upf1	PF3D7_1005500	PVX_094465	PY17X_1206900
Upf2	PF3D7_0925800	PVX_099705	PY17X_0829900
Upf3B	PF3D7_1327700	PVX_116495	PY17X_1347600
XRN1	PF3D7_1106300	PVX_098910	PY17X_0943100
RBP1	PF3D7_0414500	PVX_089680	PY17X_0716700
DCS2	PF3D7_1436900	PVX_084695	PY17X_0614400
APOBEC3G	PF3D7_1349400	PVX_083365	PY17X_1367900
Stress Granules	*P. falciparum* Gene ID	*P. vivax* Gene ID	*P. yoelii* Gene ID
Ataxin-2	PF3D7_1435700.1	PVX_084750	PY17X_1010700
eIF4E	PF3D7_0315100	PVX_095480	PY17X_0415700
Rpb4	PF3D7_1404000	PVX_086235	PY17X_1040500
SMN	PF3D7_0323500	PVX_095050	PY17X_1218200
eIF4A	PF3D7_1468700	PVX_117030	PY17X_1336600
PABP	PF3D7_1224300	PVX_123845	PY17X_1441700
eIF2	PF3D7_0322400	PVX_095115	PY17X_1219300
Other?	*P. falciparum* Gene ID	*P. vivax* Gene ID	*P. yoelii* Gene ID
RAP55 (CITH)	PF3D7_1474900	PVX_118625	PY17X_1304900
RCK/p54 (DOZI)	PF3D7_0320800	PVX_095195	PY17X_1220900
Puf2	PF3D7_0417100	PVX_089945	PY17X_0719200
ALBA1	PF3D7_0814200	PVX_123060	PY17X_1425300
ALBA2	PF3D7_1346300	PVX_083215	PY17X_1364900
ALBA3	PF3D7_1006200	PVX_094505	PY17X_1207600
ALBA4	PF3D7_1347500	PVX_083270	PY17X_1366000

RNA degradation is largely initiated through the removal of the poly(A)-tail by the deadenylation complex Caf1-CCR4-Not. In eukaryotes including *Drosophila, Saccharomyces,* and *Homo sapiens,* the core Caf1-CCR4-Not complex is conserved [92]. The various subunits of the Caf1-CCR4-Not complex functionally contribute in different ways, including deadenylation of transcripts, RNA processing, nuclear export, translational repression and feeding into the DNA damage response [91, 93, 94]. Through a BLASTp search, we identified 9 potential members of the *Plasmodium* Caf1-CCR4-Not complex (Table 8). These predicted members include the scaffold protein Not1, the deadenylases Caf1 and a HCCR4-like protein, as well as CNOT4 and CNOT3, which are responsible for ubiquitination and chromatin modifications respectively. Only Caf1 has been genetically characterized in *P. falciparum*, and genetic disruption of PfCaf1 by the piggyBac transposon resulted in mistimed expression of transcripts, abnormal expression of merozoite invasion proteins and a slight growth defect in blood stage cultures [95]. The Caf1-CCR4-Not complex is important for tasks ranging from deadenylation to ubiquitination, and may be differentially employed by *Plasmodium* to progress through its complex life cycle.

The eukaryotic exosome consists of multiple subunits and plays an essential role in RNA quality control, turnover and processing. The exosome complex has been shown to be important for 3′-to-5′ mRNA degradation. In *Plasmodium* we have found eight predicted subunits that align though BLASTP to common eukaryotic exosome components (Table 8). Rrp6 and Rrp44, which are the two active exoribonuclease components of the complex in archaeal and eukaryotic cells, are also present. An RBP (PF3D7_0903400) with putative function in exosome has been identified, which is a homolog of DDX60 in humans or Ski2 in yeast [96].

#### Transcriptomic analysis of RBPs

**Fig. 5:**
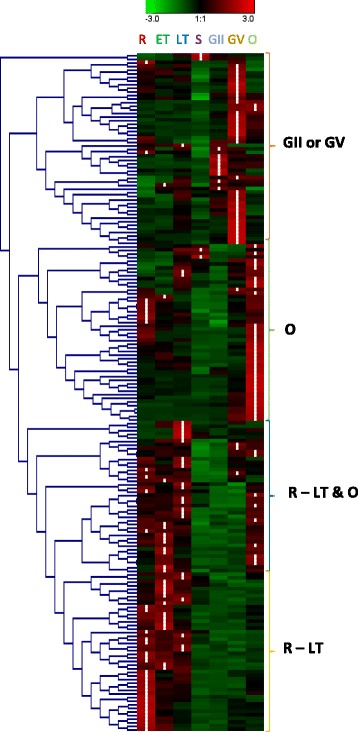
A heatmap of the expression profiles of PfRBPs throughout the blood and sexual stages. The expression profiles of the identified RBPs is provided with each gene plotted in a single row, and the experimental data for each time point provided as columns (e.g. R-ring, ET-early trophozoite, LT-late trophozoite, S-schizont, GII-gametocyte stage II, GV-gametocyte state IV, O-ookinete). Each of the similar expression-profile groups identified in hierarchical clustering is marked with braces on the right of the heatmap

Analysis of the time-course transcriptomes of RBPs during malaria parasite development revealed several interesting features [71, 97–99]. Hierarchical clustering and K-means analysis of RNA-seq data showed that 44 % (81) of RBP genes had correlated expression profiles. Their expression was detected during early ring stage, peaked at either early and/or late trophozoite, but decreased at early schizont stage (Fig. 5). Similarly, analysis of the microarray data for intraerythrocytic developmental cycle (IDC) showed that 73 % (127) of RBP transcripts were at their peak expression levels at ring or trophozoite stage. The abundance of most of the RBP transcripts (67 %, 111 genes) was suppressed during the schizont stage. This expression pattern is consistent with increased metabolic activities in trophozoites. While 27 % (51) of RBP genes showed elevated expression at gametocyte stage II or V, 44 % (81) of RBP genes had expression in multiple stages. About 24 % (44) of RBP genes upregulated during the IDC stage. It is interesting to note that several genes (PF3D7_0103600, PF3D7_0504200, PF3D7_0807100, PF3D7_1021500, and PF3D7_1307300) with putative or predicted functions in translation or translation regulators have elevated expressions during the gametocyte-stage. Confirming previous observations, PfDOZI (PF3D7_0320800) and PfDhhx (PF3D7_0807100) were found to have higher gene expression at gametocyte stage (Fig. 5). Of the 48 RNA helicases, five genes are upregulated in ookinetes (PF3D7_1459000, PF3D7_1021500, PF3D7_0821300, PF3D7_0602100 and PF3D7_0508700), whereas others conform to the general transcriptional program with reduced transcription at schizont stage.

It is noteworthy that of 28 single RRM-containing genes (Table 3), 13 are upregulated at the gametocyte stage. Noticeably, PF3D7_1126800 and PF3D7_0205700 both lack homologs in model species and showed remarkably specific elevated expression in young and mature gametocytes. PF3D7_1320900 encodes a putative peptidyl-prolyl cis-trans isomerase that interconverts *cis-* and *trans-*peptide bonds in the amino acid proline, and it was expressed at higher levels in gametocytes. A *Plasmodium* unique gene, PF3D7_1139100, showed higher expression levels at ring and merozoite stages but was virtually undetectable in other stages. Most of the 21 two-RRM containing genes (Table 3), however, had a uniform pattern of expression across different life stages of parasite development except for two genes [PF3D7_0414500 (musashi homolog 1) and PF3D7_1119800 (AFS-1)], which had notably higher expression during gametocyte stage.

Even though the *Plasmodium* transcriptome generally shows rigid, just-in-time expression patterns and ribosomal profiling demonstrates that the abundance of mRNAs correlates with their translational efficiency, many mRNAs do not fit within these bounds [100]. Therefore, assessment of RBP candidates, especially those with an enrichment of mRNA levels in a stage-specific manner merit further investigation to determine their downstream roles in gene regulation.

#### Predicted protein-protein interaction network of RBPs in Plasmodium

**Fig. 6:**
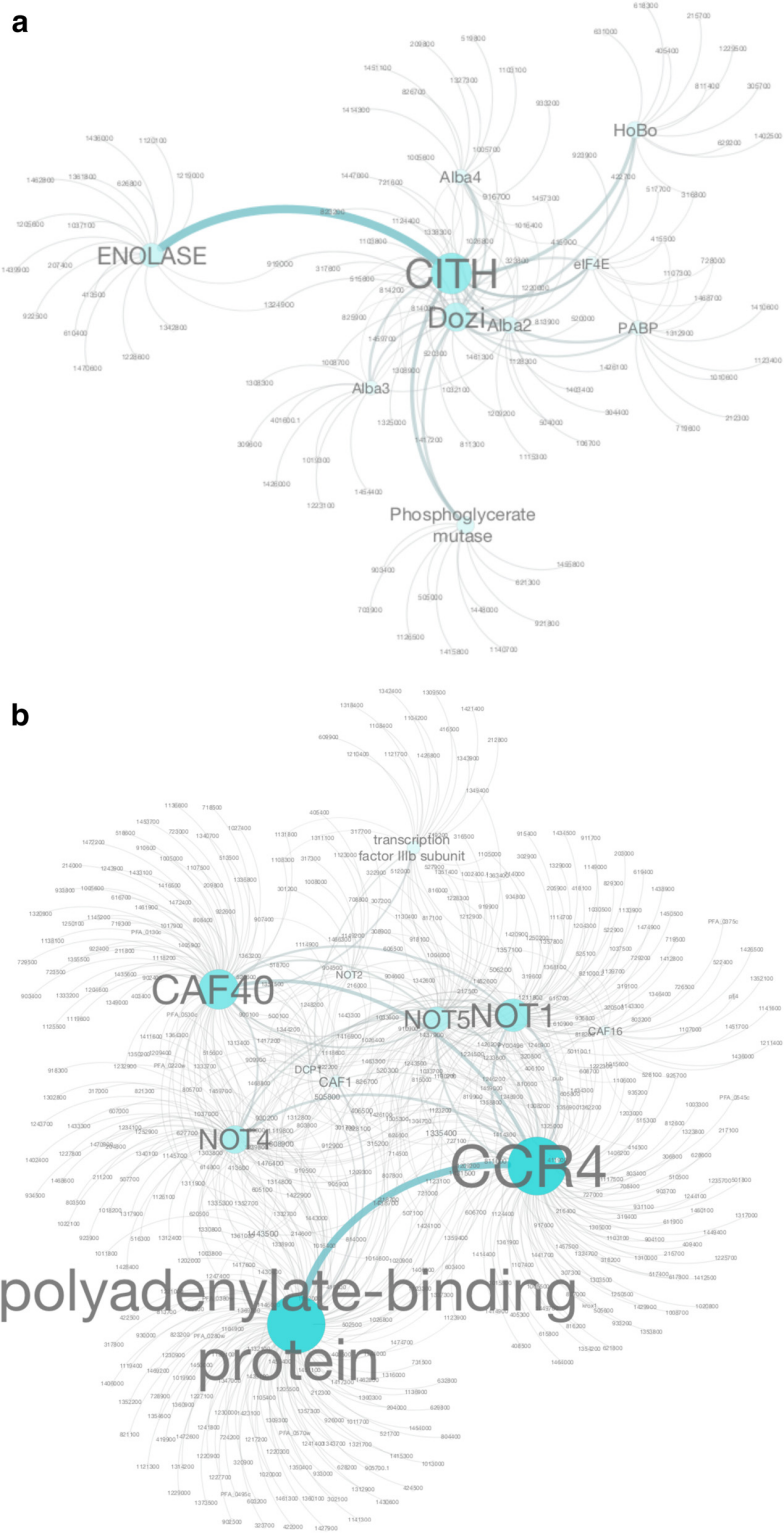
Predicted protein-protein interaction networks. **a** A bioinformatically predicted protein interaction network for the PfCITH and PfDOZI complexes. An interactome network for PfCITH and PfDOZI is provided, where protein-protein interactions (PPIs) that provide a larger contribution to the predicted network are represented with larger fonts and nodes. **b** As in Panel a, a predicted Caf1-CCR4-NOT complex interaction network for *P. falciparum* based on the PPIs found in human interactome is illustrated. The major nodes are highlighted with the functional description (for example, HCCR4). Note that these interactions warrant experimental confirmation

Because ~40 % of total *P. falciparum* genes still await functional characterization, prediction of their functions may benefit from high throughput analyses such as coexpression analysis and protein-protein interaction network analysis [101–103]. Similar analyses have been conducted with *P. falciparum*, which have proven informative [104]. Based on the available data and protein pull-down analysis of DOZI and CITH in *P. berghei* [13], we attempted to construct a protein network for the *P. falciparum* orthologs using these data along with the yeast-two-hybrid data and interactome information retrieved from the STRING database with a combinatorial search strategy including co-occurrence, co-expression and text-trimming from published literature (Fig. 6a). CITH and DOZI are two important core components of an ancient P-granule in *Plasmodium* that protect quiescent mRNA from degradation in gametocytes [13, 34]. This complex also contains Albas, eIF4E, PABP, Bruno, Mushashi, enolase, and phosphoglycerate mutase. A total of 155 interactions were mapped where DOZI and CITH topped the list with 29 and 20 interactions, respectively (Fig. 6a). Gene enrichment analysis of hits obtained from the pull-down study revealed possible direct control over cell division, glycolytic pathway and translation. To assess the evolutionary preservation of interacting partners of CITH and DOZI, we interrogated the interlogous network information available for these genes from the human counterparts. A total of 407 interactions (DOZI-350 and CITH-57) were obtained from the analysis, of which ~35 interactions were common for both human and *P. berghei*, further confirming an ancient origin and evolutionary conservation of the P-granules (Additional file 8).

Similarly, we have also constructed an interactome network for another important complex that governs post-transcriptional regulation— the PfCaf1*-*CCR4-NOT deadenylation complex (Fig. 6b). Currently there are no studies that have described the composition of this complex in *Plasmodium* species. Hence, we utilized published human Caf1-CCR4-NOT complex information to derive corresponding homologs in *P. falciparum* (Additional file 9). Following this analysis, the interologous network for human genes were extracted and the final gene set was searched against *P. falciparum* genome using BLASTp search at E-value <0.1. A total of 1090 interactions were studied, of which 774 (59 %) have homologs in *P. falciparum,* suggesting extensive conservation of interacting partners of this complex. Channeling these hits further into PlasmoDB we extracted and enriched gene ontology terms for biological processes. Most of the 774 predicted proteins of the *Pf* interactome have been categorized under primary metabolic process (GO: 0044238) that child branches into lipid metabolic process (GO:0006629), protein metabolic process (GO:0019538), carbohydrate metabolic process (GO:0005975), tricarboxylic acid cycle (GO:0 006099), nucleobase-containing compound metabolic process (GO:0006139), and cellular amino acid metabolic process (GO:0006520) suggestive of extensive interactions of the complex (Additional file 9). The entire protein network analyses in performed in this study are purely based on extrapolation of the information found in human or *P. berghei*, and hence these data presented here should be interpreted with those qualifiers.

## Conclusions

Post-transcriptional regulation is a critical way by which malaria parasite controls its developmental processes, and RBPs are basic, underpinning elements in this process. A very few number of PfRBPs have been functionally characterized through experimentation, leaving a large portion without functional assignments. About 80 % of the total retrieved 189 PfRBPs were assigned putative functions using literature search and *in silico* methods. Most of these genes are predicted to be involved in pre-mRNA processing (42 genes) and ribosome biogenesis (29 genes), and a few have functions in cytosolic granules and as translational regulators. About 50 % (25 genes) of the 42 RBPs involved in pre-mRNA processing belong to the RRM family, while 55 % of 29 RBPs participating in ribosome biogenesis are from the RNA helicase family, suggesting a large fraction of these RBP families are devoted to these two basic functions. Transcriptome analyses of RBPs show both stage-specific enrichment of transcripts and mixed-curve expression profiles suggesting involvement of complex cues in their regulation. Some of the components of pre-mRNA processing and ribosome biogenesis, which are thought to be essential for these basic processes, show stage-specific enrichment of mRNA levels. Because most PfRBPs have no experimentally defined functions, these data may provide a guide to prioritize a subset of genes with an aim to better understand the basic biology of the parasite.

## Methods

### Database search for sequence retrieval

A multipronged search strategy was employed to retrieve putative homologs of RNA-binding proteins (RBP) genes from public domain databases. Initially, a ‘text’ based search was performed against PlasmoDB Version 12.0 (http://plasmodb.org/plasmo/) [105]. For example, to identify RBPs with a zinc-finger (Znf) like domain, “RNA-binding” followed by “Zinc finger” key words were used. Similarly, RRM, RNA helicase, Puf, K homology, Alba, PUA, S-1, YTH, PWI, SWAP, G-patch key words were used in quotes to search for RNA recognition motifs, RNA helicase, Pumilio-Homology Domain, K homology, and Acetylation Lowers Binding Affinity, pseudouridine synthase and archaeosine transglycosylase domain, S-1 motif, YT521-B homology, PWI, Suppressor-of-White-APricot domains, and G-patch motif domain containing genes, respectively. As a second strategy, a hidden Markov model (HMM) for each of the RNA-binding domains was constructed using a reference set of genes annotated from the “text” based search using hmmbuild in package HMMER version 3.0 [106]. Multiple sequence alignments were performed using the MUSCLE program using default parameters [107]. The created HMM profiles were subsequently used to perform hmmsearch (http://hmmer.janelia.org/search/hmmsearch) against the *P. falciparum* genome. As final strategy, Pfam ID’s of each of the putative RBDs (Additional file 1) were used to search PlasmoDB. The genes retrieved from each of the above analyses were combined and parsed to remove duplicate genes that were retrieved in multiple search strategies to arrive at the final list of putative RBPs.

### Domain mapping and confirmation

To define the protein domain organization of the putative RBPs, sequences were subjected to domain profiling using the Simple Modular Architecture Research Tool (SMART) [108] and Conserved Domain Database (CDD) search tools [109]. While the SMART searches use the underlying SMART database, which consists of manually annotated protein profiles [110], the NCBI-CDD search hosts multiple databases, including CDD profiles v3.13. In addition, the CDD database uses protein 3D models in conjunction with primary sequences to classify domains into different superfamilies [109]. Where possible, a superfamily of each identified domain was used to predict RBP function in addition to annotations derived from homology searches (see below).

### Functional annotations

Functional assignment of the genes predicted to encode RBPs was achieved by combining results from existing annotations from PlasmoDB v. 12.0, protein BLAST (search of GenBank [111], literature searches, and domain superfamily classification from CDD searches. BLASTp was carried out against the reference sequences of five selected model organisms—*Saccharomyces cerevisiae* (taxid: 4932), *Caenorhabditis elegans* (6239), *Arabidopsis thaliana* (3702), *Drosophila melanogaster* (7227), *Homo sapiens* (9606) and *Trypanosoma cruzi* (5693) using the following parameters: word size-3; Blosum 62 substitution matrix, gap opening 11 and extension 1. Because *Plasmodium* genes are often interspersed with low complexity regions (LCR), BLAST searches were configured to negate the impact of these regions on the outcome by selecting LCR filters in algorithm parameters. To avoid false functional assignment due to partial sequence matching, we employed reciprocal searches against *Plasmodium* genomes using sequences from model species or Trypanosomes, and more stringent criteria (≥40 % identity of the query protein and covering ≥80 % of the target gene) to assign specific functions to the proteins. In certain cases, the criteria were relaxed if the orthologs from more than one model species had a similar functional assignment, and when protein homology extends beyond the functional unit of the query protein. In the event of lack of homologs in models species, a relaxed modified-search was performed with lowered E-value (e.g. 10) and its use is noted where it is applied in this study.

### Multiple sequence alignments and phylogenetic reconstruction

All multiple sequence alignments made in the study were performed using MUSCLE software with default parameters (gap opening and extending penalties as −2.9 and 0) as implemented in MEGA version 6.0 [112]. Similarly, all phylogenetic reconstructions and molecular evolutionary analysis were conducted using MEGA v6. The genetic distances were estimated using Poisson correction and phylogenetic trees were constructed following Neighbor-Joining method [113]. Tree robustness was evaluated using 1000 bootstrapped replicates.

### Homology modeling

Three dimensional structures and domain folds of proteins are commonly more conserved than the amino acid sequences themselves. Hence, in this study we threaded 3D models for either defining different classes of RBPs, or to locate conserved residues, or to differentiate prokaryotic vs eukaryotic protein structures. A representative homology models for each of the five major RBDs (RRM, RNA helicase, KH, Puf, and Alba) were constructed by structural threading using algorithms implemented in I-TASSER (Iterative Threading ASSEmbly Refinement) [114] or Swiss-model [115]. The Swiss-model server automates building the homology model by first searching for a suitable template for constructing a reference-based model. Following this, the model was subjected to strained angle correction, and quality control parameters were estimated (e.g. Qmean Z-score, a likelihood of comparable quality of an estimated model to the native structure [116]. Similar to Swiss-model, the I-TASSER server also automates the model building, however, it uses three different conventional 3D model building procedures to do so (homology modeling, sequence threading, and *ab initio* modeling) [114, 117]. The procedure uses C-score and TM-score as quality parameters to estimate the model quality [114, 118]; where C-score is a confidence score (−5 to −2.25, higher is better) while TM-score (0–1, a higher value translates to increased confidence in the model) measures degree of absolute similarity between the built model to the native structure [114].

### Transcriptome analysis

Transcriptome analysis on putative RBPs was performed using curated microarray and RNA-seq [119] datasets downloaded from PlasmoDB. Heat map and clustering of the RNA-seq data was performed using the MeV software [120]. Average linkage agglomeration rule was applied to cluster genes hierarchically with similar expression patterns. We also combined self-organizing maps data to the hierarchical clustering to derive stage-specific gene expression, which was determined using 2000 iterations at α-0.05.

### Interactome analysis

An interactome analysis for PfCITH and PfDOZI was performed based on published protein-protein interaction (PPI) data for the orthologs of these proteins in the rodent parasite *P. berghei* [13]. The top six hits that have assigned putative functions in PlasmoDB were further used to search the STRING v9.1 database for identifying interacting partners. The STRING database reposits known and predicted protein-protein interactions. Known interactions are confirmed physical interaction between proteins, while predicted interactions were derived from four sources: genomic contexts, high-throughput experiments, coexpression and literature review [121]. We used a high-confidence score (0.7) to select the most likely interactions for further network construction using Cytoscape (www.cytoscape.org).

We have also constructed an interactome network for the PfCaf1*-*CCR4::NOT complex associated genes using human homologs. Following this, PPI data for human homologs were retrieved from Interologous Interaction Database (http://128.100.137.135/ophidv2.204/ppi.jsp) and the hits were used to collect *P. falciparum* homologs using BLASTp search against PlasmoDB with E-value <0.1. Interactions for each of the core components were searched for gene ontology terms in PlasmoDB and enrichment for biological process and primary metabolic processes were done.

## Additional files

Additional file 1:
**A comprehensive list of putative RNA-binding proteins retrieved from** ***Plasmodium*** **spp.**: This summary table shows each of the putative RNA-binding proteins retrieved from *P. falciparum* and provides their Gene ID, their protein length, orthologs in *P. vivax* and *P. yoelii*, search results against model species, functional annotations, domain organizations, structural similarity to homologs, and Gene Ontology (GO) terms. Blue, pink, and green boxes are used to denote transmembrane, low complexity, and coiled-coil regions respectively, and NH for no homologs. Naming of *Plasmodium* genes is tentative, and should be used cautiously until further evidences are available about their functions. (XLSX 2203 kb) Additional file 2:
**A multiple sequence alignment of the 122 RRM domains found in 66** ***Plasmodium falciparum*** **proteins.** The Gene IDs for proteins predicted to contain highly conserved RRM motifs (RNP1 and RNP2) is provided along with the predicted secondary structure (mapped to the top of the alignment). Please see the legend key provided at the end of the alignment for meaning attached to color-code resides and letters used for writing consensus sequence. (PDF 291 kb) Additional file 3:
**An expanded functional description and 3D model of the RRM domain in** ***Plasmodium.*** (a) A representative 3D model of a RRM domain constructed using PfPABP3 (PF3D7_0923900) as a query and PDBID: 2jwn as a template using default parameters as described in the Materials and Methods section. The canonical RRM fold is marked on the 3D model, where L-stands for link; h-helix, 1–4 β-sheet, and RNP1&2 are conserved features of RRM domain. (b) A categorization of putative functional roles of RRM motif in *P. falciparum* and their associated genes. (PDF 75 kb) Additional file 4:
**A list of Poly(A)-binding proteins retrieved from** ***Plasmodium*** **genomes along with their putative cellular locations.** (PDF 63 kb) Additional file 5:
**Multiple sequence alignment of DExD/H RNA helicases from** ***P. falciparum.*** All the conserved motifs representing helicases are mapped to the multiple sequence alignment. For residue color and meaning of consensus letters please see key at the end of the alignment. (PDF 336 kb) Additional file 6:
**A phylogenetic representation of the RNA helicases found in** ***P. falciparum.*** Phylogenetic reconstruction of 48 PfRNA-helicases show uniformly higher support for all the tree branches, which is suggestive of deep-evolutionary conservation at the sequence and probably at the functional level. Two subfamily clusters representing two functions, small subunit rRNA helicases (SSU) and pre-RNA processing (Prp) have monophyletic representation. (PDF 57 kb) Additional file 7:
**A structural and sequence-based comparison of PfPuf1 and PfPuf2.** (a) A predicted model of PfPuf1 (using PDBID: 4dzs as a template) superimposed on a predicted model of PfPuf2 (using PDB ID: 3 k49 a template) at 3.4 root mean square deviation confirming the signature concave structure common to PUF domains. (b) A multiple sequence alignment of predicted PUF-domain sequences. PfPuf1 and PfPuf2 are 25 % identical at the domain level. (PDF 233 kb) Additional file 8:
**A unified gene list identified as common between human and** ***P. falciparum*** **DOZI interactomes.** Gene IDs from *P. falciparum*, along with their predicted function and biological process are provided. (PDF 49 kb) Additional file 9:
**A list of Caf1-CCR4-NOT complex genes identified by searching against human PPI database.** The interactions were further used to perform BLASTp-search against PlasmoDB at E-value <0.1. (PDF 41 kb)
