# Supplementary material for: A bioinformatic survey of RNA-binding proteins in Plasmodium
Source: BMC Genomics. 2015 Nov 2;16:890. doi: 10.1186/s12864-015-2092-1 (PMC4630921; doi:10.1186/s12864-015-2092-1)
Supplement: Additional file 2: — A multiple sequence alignment of the 122 RRM domains found in 66 Plasmodium falciparum proteins. The Gene IDs for proteins predicted to contain highly conserved RRM motifs (RNP1 and RNP2) is provided along with the predicted secondary structure (mapped to the top of the alignment). Please see the legend key provided at the end of the alignment for meaning attached to color-code resides and letters used for writing consensus sequence. (PDF 291 kb) [file 12864_2015_2092_MOESM2_ESM.pdf]

Additional file 2

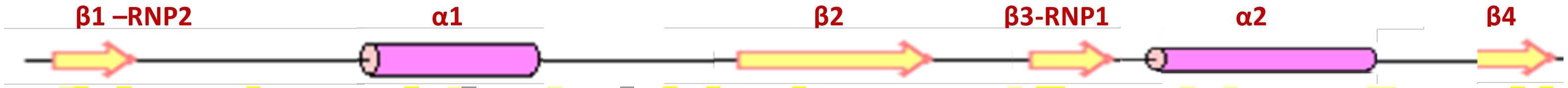


```
PF3D7_0414500-RRM1   KFFIGGI----P-QYIT---SKH---ITEYFE---QY----G-TVQHVV(1)-AQDH-ETKRNR(1)-FAFVTMASHINKERILR-----DTHEL---NGK-RVDVR
PF3D7_0414500-RRM2   KIFVGGL----N-YYWT---KDT---LESYFS---TF----G-EIDVVQ----IVLD-SSGRSR(1)-FGFVVFSNENSVAKVLK----HKRHKI---YDK-MVEVR
PF3D7_0517300-RRM1   RIYVGNL----P-SHVS---SRD---VENEFR---KY----G-NILKCD------VK-KTVSGA(1)-FAFIEFEDARDAADAIK---EKDGCDF---EGN-KLRVE
PF3D7_0517300-RRM2   VVEVTGL----P-ISGS---WQD---LKDHLR---EA----G-ECGHAD----VFKD-------(1)-TGEVSFFNKDDMLEAID---KFNGSIFRSHEGE-KSKIS
PF3D7_0606100-RRM1   VVYVKNL----S-SDIT---EEN---IREKFG---SC----D-EIISIT-----FKN-FPGLNQ(1)-YCQIEFKTSEGITNASR----LNGESL---LNV-PMVVS
PF3D7_0606100-RRM2   IVYMENI----P-EKYG---EED---IKAFFQ---NV----G-NTTSYK----LQYN-EQKKVH----TAFVEFKNEEHAKAALN----LSGTKV---GLH-EICIR
PF3D7_0606500-RRM1   VLLLKNV----P-EKVD---EED---IISFMR---PF----L-RNKNPE----IIFD-------(2)-DIIVKLYDDELIESIYT---YFNEHPTQI-KGS-FVKVK
PF3D7_0606500-RRM2   LVSVINL----H-YPVD---IEL---IYYLFS---KC----G-TVEKII----TFSR-NPVLYQ-----ALVQFDNVETAKEAIK---TLHNRNIY--DGCNTINIQ
PF3D7_0606500-RRM3   VLICYNI----PKEYTD---VNK---LFNLFS---IY----G-FVTRIK----ILRE----KPD----AALIQYSNYIFSSLAQE---YLQRARI---SNQ-SIEVN
PF3D7_0606500-RRM4   CLFISNL----N-ENVN---EDC---VMNLFN---KY----G-NINKLQ----FLPV-KEGKRH(1)-SIIVEMNTEDMATKALM---DLHNFYL---KDR-YIKVS
PF3D7_0629400-RRM1   TLWVGDL-EKIK-DEVV---DEN-YILYCMFY---EF----AEDIIRIK(3)-EKSN-QK---N(1)-YAFIEFSTYEVAKYCFE---KLNGKWI---PGK-AHRFK
PF3D7_0629400-RRM2   SIYVGSL----P-INTT---KEE---IENLFC---NI----YNSICFVK(1)-IKNT-QKSPHK(1)-YCFIHFFNYDECLRALT---EMNGYIF---KGC-KIKVS
PF3D7_0823200-RRM1   KIFIGSI----P-KDVT---EEE---LKTEAA---KY----G-TITQVY----YVPA-TAQSPR(1)-WAFITYEQRSEAYKAIE---ALDYKCIFPNSQR-PLDVR
PF3D7_0823200-RRM2   NVFVFHL----P-SHWT---DME---LYQHFQ---HF----G-YVVSAR----IQRD-ANGRNK(1)-YGFVSFNNPESALNAIK---GMHGFYV---SGK-HLKVQ
PF3D7_0916700-RRM1   RLMVRNI----P-FSTR---DEQ---FLKYFE---SF----G-EIEDGI----IVRE-KEGRSK(1)-YGFVTFKYVESVQKCLK-----SSHTL---DNK-ELQVR
PF3D7_0916700-RRM2   KLFVRNL----S-QKTN---VST---LRSIFE---KY----G-KLEECV----IIHD-NEGKSK(1)-YGFLTFSSPREAFKVMQ----QPERII---DNR-VVFLH
PF3D7_0920900-RRM1   KLYVKNI----N-EHIT---KEN---FESYFS---KI----DGYIETRF----VVDT-SRTSRK----FAYIDFENKTKALAFLH---TLQNSNI---ENFKTLTLN
PF3D7_0920900-RRM2   TVVVKNL----H-FNTR---KNK---LQNIFG---QI----G-EIENIYLSKKISEN-NIKRNK(1)-FAFITFKNSNDATSSL----ILNDIII---DGR-NILIS
PF3D7_0929200-RRM1   SIIISNV------NSKN---LEL---YKKEFS---KF----G-KIYNIY---------HDNEKK----ITYVKYDNKNSCDTAIS---TMNNQTI---DGD-TITII
PF3D7_0929200-RRM2   TIIVTNV----P-TYLN---AED---IFSAFQ---ET----G-KIIDVQ----ILMN-EKRKLT(1)-IVSIEYEKNESASDAVR---MYDGGFL---NDN-RIRVF
PF3D7_0935000-RRM1   TLYVNNL----E-DKIN---VND(4)LYEFFC---PY----G-NIIDIN----IKKS-NK--GR(1)-QAFIVFNNIASSTLAYK---NLKGKMF---LKK-NININ
PF3D7_0935000-RRM2   TLLVQNL----P-DEIN---KNA---LEILFN---QY----P-GFYEVR----YIPG-KN---------IAFVDFTAQEHAEISMT---GLQNFKI---TPHHPMKIS
PF3D7_1004400-RRM1   KIFVGNI----S-SSAR---EED---VRRRFE---QY----G-DIMHMQ----WKKR-----------FAFIEYYKASHALNALE---KENGKMF---FGE-ELSVQ
PF3D7_1004400-RRM2   RIVVKNI----D-EKAS---WQD---LKDFGR---EV----G-SVSYAN----IVDD-YHSKEK----FGIIEFYNHENAKDAIN---ILNGKSF---YGR-SVDVI
PF3D7_1006800-RRM1   RVYVGNL----P-WKVT---WPI---LKNHMK---KA----G-DVVRVD----IFED-TQGRSK(1)-CGIVEYATYEEAQEAIS---SLNDSKL---EDR-LIFVR
PF3D7_1006800-RRM2   TLIVYNL----P-PQVT---WKE---LKDLFR---KH----G-RVVRAD----LKNEDNSSKEL----IGVVIMENEYEAKNAID---ALNFCNF---DGY-ILKVN
PF3D7_1022000-RRM1   NIFLGNI----P-PNIT---EER---LKNVLE---IF----G-YIIHIE----YKWS-ID--KW(1)-YAFVYFIDEKCAINAVN---FLNQKKF---FDN-SPNHK
PF3D7_1022000-RRM2   NLFLYGI----P-LKWT---ELN---LIQLVN---KY----G-HVVGLR(4)SKEND-KKQGNR(1)-FGFVSYDNKKSAIEAFE---ELSKMYI---HGK-LLKVQ
PF3D7_1022400-RRM1   CIYVGNL----P-GNVI---EEE---VYDLFG---KY----G-RIKYID----IKPS-RSSSSS----YAFVHYYDLKDADYAIE---RRDGYKF---DGF-RLRVE
PF3D7_1022400-RRM2   RVIVTNL----P-DNCR---WQH---LKDIMR---QC----G-DVGYAN(1)----------ER(1)-KGIVEFVSYDDMLYAIE---KFDGAEFKVYDD--VTNIK
PF3D7_1107100-RRM1   RIFATRL----P-FEAS---KKD---LEKYFS---KF----G-KIVDIY(1)-SRNI-SNNKNK(1)-FGFVSFEKQESMNKVLK----EKLHII---CGK-EIVVD
PF3D7_1107100-RRM2   KLFVTKL----N-SATT---IEK---LRNYFE---KF----G-EIIDIY(1)-PNDV-YTNRPR(1)-IAFVTFLDNESVKNILS--DEHSKHII---DGK-EVVLD
PF3D7_1217200-RRM1   RLIIKNI----P-KYMN---EID---LKKHFF(7)NF------QITDIK(6)IIKNK-EHYESR(1)-ICFIGFLNNYHCQCFKK---FFNNTYI---NTS-KIIIE
PF3D7_1217200-RRM2   KLIIFNL----P-P-IN---EQD---IKSLCE---RY----G-PIVDVK(49)DDYN-KKDDNN(32)YAFVNFMFPSSCEKAKI---HLDNKIY---RGK-ILIAK
PF3D7_1217200-RRM3   TIIIKNL----S-IYTN---QND---IINLFK---QY----G-ILKRVS---------FSPYNN----ICIIQYENADNAKKAF----ISNSYIR---YKKLPLYLE
PF3D7_1217200-RRM4   SIYIKNI----N-FNTK---EED---LKNLFD---KM(4)TC-NIVKSK(12)ISNQ-YNTVSS(1)-YGFAEFKNKELAMEAIK---RLTGTRL---NDH-LLEMS
PF3D7_1217200-RRM5   KLVVKNL----A-FQVN---KEE---LRKLFS---AF----G-NVKSVR----IPKN-VYNRSR(1)-YAFIEFMSKKESCNAIE---SLQHTHL---YGR-HLIID
PF3D7_1224300-RRM1   SLYVGDL----N-EDVT---EAV---LYEIFN---TV----G-HVSSIR----VCRDSVTRKSL(1)-YAYVNYHNLADAERALD---TLNYTNI---KGQ-PARLM
PF3D7_1224300-RRM2   NIFVKNL----D-KSID---NKA---LFDTFS---MF----G-NILSCK----VATD-EFGKSK(1)-YGFVHYEDEESAKEAIE---KVNGVQL---GSK-NVYVG
PF3D7_1224300-RRM3   NLYVKNF----P-DSVT---ETH---LRQLFN---PY----G-EITSMI----VKMD-NK--NR(1)-FCFINYADAESAKNAMD---NLNGKKI---TDDGQIDET
PF3D7_1224300-RRM4   NLYIKNL----D-DGID---DIM---LRELFE---PF----G-TITSAK----VMRD-EKEQSK(1)-FGFVCFASQEEANKAVT---EMHLKII---NGK-PLYVG
PF3D7_1306900-RRM1   TLYIKNL----N-DRVK---TDE(4)LKDLFN---TY----G-EIKDLI----VMKS-FW--RK(1)-QAWVVYDDKECATKALN---ALQGYVL---FGK-IMQIN
PF3D7_1306900-RRM2   ILFVENV----V-ENVD---TQA---FNDLFK---NY----A-GFVEAR----IIPQ-RN---------VAFVDFTDETTATFAMK---AVQNYEL---QGS-KLKIS
PF3D7_1321700-RRM1   TVLVLNL----D-LKAD---ERD---IYEFFS---EV----AGKVRDIQ---CIKDQ-RSGKSK(1)-VAYVEFYTQEAVIKALA----ANGMML---KNR-PIKIQ
PF3D7_1321700-RRM2   KLYIGGL--LGPLSNIT---EQE---LKQLFN---PF----G-DILDVE---IHRDP-YTGKSK(1)-FGFIQFHKASEAIEALT---VMNGMEV---AGR-EIKVG
```

```
PF3D7_1321700-RRM3    NLVLSNM(5)EN-IGSD---PDF---FNDILE(7)KY----G-KVVNIW-----LDT-KN--ID(1)-KIYIKYSNNDESLKSFQ---FLNGRYF---GGS-LINAY
PF3D7_1359400-RRM1    KLFIGRV----P-KNIE---EDQ---LRPIFE---EY----G-IVNEVV---IIRDK-ITNVHK(1)-SAFVKMASISEADNAIR---LLNNQK--------TLDAQ
PF3D7_1359400-RRM2    KLFIGSL----P-KNIT---EDN---IKEMFS---PY----G-TVEEVF----IMKDNSTGLGK(1)-CSFVKFSYKEQALYAIK---SLNGKKTLEGCTR-PVEVR
PF3D7_1359400-RRM3    NLFIFHV----P-NEWQ---QTD---LIQAFS---PF----G-ELLSAR----IATEKNTGRNR(1)-FAFVSYDSLESAAAAIS---QMNGFMA---LNK-KLKVT
PF3D7_1360900-RRM1    SIFVYNF----P-NEWM---END---IKKNFM---IF----G-TINNII----IDKD-INI-------YAFIQYNDTEASQKAIE---VMNGKEI---NGK-LLKVT
PF3D7_1360900-RRM2    TLFVFYL----P-PHWN---DQD---LFDKFK---TF----G-NLESAT----VAKK-NDKTSK(1)-YGFVVYTDPHSAALAIS---NMNKVEVY--TGK-RLKVL
PF3D7_1360900-RRM3    TIFVFYL----P-NDWS---DKD---LKRHFS---HY----G-NILGAT----IKRE-TNGKSR(1)-YGFINFENQQSAINAVA---GMNGFNA---GNK-YLKVS
PF3D7_1409800-RRM1    RIYVGNL----P-SHVS---SRD---VENEFR---KY----G-NILKCD----VKKT-VS--GA(1)-FAFIEFEDARDAADAIK---EKDGCDF---EGN-KLRVE
PF3D7_1409800-RRM2    VVEVTGL----P-ISGS---WQD---LKDHLR---EA----G-ECGHAD----VFKD-------(1)-TGEVSFFNKDDMLEAID---KFNGSIFRSHEGE-KSKIS
PF3D7_1420000-RRM1    TLYIANL----D-AQVD---EEI---LCELFM---QC----G-NVKNVH(1)-PRDK-INGYHA(1)-YGFVEYEYEYECEYAAN---ILNMTKL---FGK-ALRCN
PF3D7_1420000-RRM2    NLFIGNL----D-DEVD---EKM---LFDIFS---SF----G-QIMTVK----VMRN-EDDTSK(1)-HGFISYDNFESSDLAIE---NMNNQFI---CNK-KVHIS
PF3D7_1468800-RRM1    KLYIGNI----P-PNSK---QED---VVDFFN(5)VI(4)LDVKIGDVQ(4)IKCEI-FNSDSR----FCFLEFRTVQITWLCLK----LDSIPY---NNY-CLRIG
PF3D7_1468800-RRM2    RLYIQNL----P-HDLK---DEQ---IKDLLE---QF----G-DLKAFN(1)-IKDL-NTGLNK(1)-YGFFEYEDSSCTQLAIH---ALNGFVC---GQN-ILNVK
PF3D7_1468800-RRM3    DLIVDSQ------YEEI---LKE---VKEEAE---KY----G-TLQNIV----IPKP-NKDLSY(5)-KIFLHYADEATARKAQY---MFNGRLF---EKR--VVCA
PF3D7_1236100-RRM1    KLHIQNI----P-PHIT---DVH---LRSLLG---NV----G-FIKDIC(6)KQMNN-NNFANKKIYNTALVTFNTHEEALNVLK---NIKNLIDTSGEER-NIDAK
PF3D7_1236100-RRM2    NLFIFHI----P-SEWT---DLD---LFQHFC---CF----G-NIISSK----IQRD-STGRNS(1)-FGFVSYDNVISAQHAIQ---FMNGYFV---NNK-YLKVQ
PF3D7_1405900-RRM1    KVFVRNI------KESD---VPL---ITKEFE------------RICNKH----FFFS-NNSYTS(1)-NAICHFKSAKDAEDFIS---KYNNIKL---CNS-YIKSE
PF3D7_1405900-RRM2    KVILQNL----S-RSCH---VEN---IQKLFK---HI------EPNAKI----FFPK-KNNKKQ(1)-YAIVSFASLYNAQKALQ----LNKTKL---CGN-IITIQ
PF3D7_1405900-RRM3    TLFITNI----P-PETT---DDE---IRNYII--NNI------NKDYIY----IKTC-QSNNKN(1)-SVFVKLKYKQDADTFLK---KIGEYEY---DEN-NDEND
PF3D7_0723900-RRM1    EIILLNI----Y-LELS---NDH---LEMLLK---IF----G-TIEKIY----MDDN----EGV----HAI--YTSLSSAKKAKE---FLDNLKI---KNR-RMQVI
PF3D7_0723900-RRM2    CLWVGNI----L-KNYFFNTANI---LKTMFS---YF----G-EIRNIK----YVND-KN--------CFFLQYKNVESAINARN---HMFGIQI---SKSTILNID
PF3D7_0728900-RRM1    SIYITGL----P-NDVV---KEE---IYEVFK---KA------GIIKID(8)KIYYD-DNNNIK(1)-DALVTYVYTQSVDMAIK---YFDNFLF---RQNCIIHVE
PF3D7_0728900-RRM2    IVVFRNV---FSYEDAM---KYD(4)FYEFIK---NM(4)IK-KYVPVH----KVYP-IPKHPH(1)-IVCVKFKGVEEAETVVS---CFNDIEL---NGK-KLEVY
PF3D7_1020000-RRM1    TIFVGNI----PIKDVS---ISK---LLKILN---IE----KSIVETVR(8)KYADK-KRLGVM(1)-KKFTDVKDNKNALVTLK(10)RNGTVY---EGY-VLRVN
PF3D7_1020000-RRM2    SICIKNL----C-KKLN---EKD---LYEIMK---DV----D-TIKGVR(1)-LRDT-ATSMST(1)-TAFILFESRSAVKKAIQ---QFNGYTI---NDR-QIVVE
PF3D7_1119800-RRM1    RLYVGNI----P-GSAT---RQE---LIKIFE---EY----G-KISDID----IKYN-RNSNGT(1)-YAFIEYENPKSAEKTIQ---KRNGKKF---KGY-MLKVE
PF3D7_1119800-RRM2    RVVVKHF----PRFFKN---------IKEFLS---RA----G-KVLYIH------------KDN(1)-LIIAEYEDKESMIKAIS---TLDRTIY---NSKRKVYVR
PF3D7_1326300-RRM1    RLYFGNL----P-LHLG-LSENA(4)VWNEMK---KR(4)DE-NINPVL----YVWF-AKDKGN----YGFVEFATVEETERALT----MDGMIC---KGV-ALKVS
PF3D7_1326300-RRM2    IVSLESI----NHEEYS-TILED---IKEGFH---SQ----G-LIINAI----LINQ-KYVQNT(5)-DVIIEFESEDSVDKSIQ---NMSSRKY---EGK-FIKMD
PF3D7_1402700-RRM     NLYLGNL----S-PEVT---EEY---LCQKFG---KF----G-KVNSVK(5)KDEDK-KK--AR(1)-SGFVCFENIEDAENAKD---ALDGVEM---CGN-IIRIG
PF3D7_0319500-RRM     TVYIGNL----S-IYTT---QQQ---IYEHMS---KA----G-DVENII(1)-GLHR-TEKSPC(1)-FCFVVYKKKEGYTQAVN---FLNNSIL---DGR-IIRVD
PF3D7_0416000-RRM     NIIVTNI----P-KDLS---AQE---IMETFK---CV----G-NVLGAD----IMLT-SKGTHS(1)-RACITFPDFESASLAAS---QYDGGTL---NNQ-KIKVF
PF3D7_0503300-RRM     SLLIRKL----K-FDTS---PSI---VREKFK---RF----G-AIKDVY---LPIDY-YTKEPR(1)-FGFVEFYDAKDAEQALK---EMNGSEI---DGS-RIEVF
PF3D7_0515000-RRM     SIFINNI----NQVPVI---EKI---LYEEFL---PF----G-NIEYVR----YIPN-KN--------IAFIQFTNRVNAEFAKI---AMSDQPIEN-YST-ALTIK
PF3D7_0615700-RRM     RVKISNL----D-YTIS---KND---LMELFS---NV----C-KVVNAW----INYD-HTDRSN(1)-TAVCVFENINDAQKAID---KYDGSEI---EGL-SIKME
PF3D7_0716000-RRM1    ELYVGNI----P-QHID---IQE---IVKYLN---SC----L-LILYNK----ENEN-ENICLK(8)-YAFVEFRNIQDTSNCM----LLNGINF---YGN-NLRIG
PF3D7_0716000-RRM2    KLCVSNI----S-KNND---TSK---IKELLE---AF----G-EIKNFE----FFYG-DETSDT(1)-ISLVEYVNTENAIQAHK---ILNQNTSYKIQFE-HEIIN
PF3D7_0716000-RRM3    VIVLNKI----A-TFEELSDSSE---YKDIVE(7)KY----G-KTLEVV(52)DDNN-NDSNDN(54)CAFIHFENIESATKARK---ELSGRKF---GAN-IIEAN
PF3D7_0812500-RRM     ILFVCKL----N-PVTE---EED---LKIIFS---RF----G-NIKSCK----IIKDKVTNNSL(1)-YGFIEFEKKEDCLNAYF---EMDNVVI---DDR-RIHVD
PF3D7_0815600-RRM     TVRVTNL----S-EDVN---ENE---LSNLFG---KV----G-NIVRMF----LAKHKETQNSK(1)-FAFITYSKREEAKRAIE---KLNRHGF---ENL-LLSVE
PF3D7_0923900-RRM     SIFVGNV----D-YSTQ---PEE---LQSLFS---EC----G-LINRVT(1)-LVNK-NTGHSK(1)-YAYIEFADASSVRTALS----LSESFF---KKR-QIKVC
PF3D7_0933000-RRM     SLWIGNI----P-FDIT---ENE---LYEILC---KV----G-VVRNVR(1)-KYDV-DKNMSK(1)-FAFCEYKDVETCLLAFK---YINGYEI---KGR-KLKVF
PF3D7_1002400.1-RRM   TLYVSNL----S-SKIT---TAK---LQDIFE---KY----G-NIEKCY----VISNPITKESR(1)-FGFVTFNNSEDAENAMN---KANKMEI---EGR-EINVE
PF3D7_1002400.2-RRM   TLYVSNL----S-SKIT---TAK---LQDIFE---KY----G-NIEKCY----VISNPITKESR(1)-FGFVTFNNSEDAENAMN---KANKMEI---EGR-EINVE
PF3D7_1024200-RRM     RLKLRGL----P-FDAS---EEE---IKNFFR---DF------QLTKQAYPIHIIKG-IKNKPT(1)-HAYVYFDDEEEARNACQ---AMNRKYI---RDR-FVEIY
```

```
PF3D7_1119300-RRM      EVLDKAA----DHFEEF---YEE---VFDELM---KY----G-EIEDMV----VCDN-IGDHII(1)-NVYIKYTHEDYAEKAVN---ELNGRFY---AGK-PLQIE
PF3D7_1126800-RRM      SILIRNL----N-YDTS---PDK---VRKIFE---NV----G-KVKDVY---LPLDH-YTRKPR(1)-FGFVEYFESKYAKEAIN---ILNHSRI---DGN-EIRII
PF3D7_1139100-RRM      KLFFGNL----A-PITT---EKD---MHNLFS---NF----G-RCDSLI----ILKD-RRSKSR(1)-SGFVTFYNRDEAVNAIK---CLNNKIILSGAHK-PLEVR
PF3D7_1207500-RRM      YLFICNL----D-NRLS---SKD---VTHFFN---YF----V-GIDNCI-AKIKKNR-FTGRNM(1)-HGILKFKKPCDATLVLL---NYQGIKL---GDK-NIILT
PF3D7_1224900-RRM      ILYVRNL----P-YKIS---ADE---LYDIFG---KY----G-TVRQIR------KG-NAEGTK(1)-TSFVVYDDIYDAKNALD---HLSGFNV---AGR-YLVVL
PF3D7_1248200-RRM      KICIQGI----S-ESVS---QAN---IKECFK---KF----G-DIKSIK----VIPK-DS--------KMFISYSNSQAAKKASD--KYKDGLLL---NGC-NLTVH
PF3D7_1330800-RRM      KIILHHT----S-KDTS---QQD---IRNMFE---QY----G-YVVEVF----FPKT-YP--------KVFVRFDNLQSCIKALE----QDGCLL---KDK-YIQVT
PF3D7_1360100-RRM      RIFCGNL----G-NEVS---SDI---LANAFR---KY----K-SFNMAK(1)-IRDK-RNNKTK(1)-YGFVSLSDPQDMLDALK---TMNNKFI---GNR-PITVK
PF3D7_1367100-RRM      TLFIGRL----S-YEVS---EQK---LKKEFE---SY----G-KIKTVK----IIYD-KNLKPR(1)-YAFIEFEHTKSMNDAYK---LADGKKI---ENR-RILVD
PF3D7_1406000-RRM      IIIITNI----H-GEAR---DDY---IKEVFE---RF----G-QIKNLH(1)-NIDR-RTGFLK(1)-YAFLEYENFVDAKRAID---EMDGTML---LNQ-EIHVD
PF3D7_1445600-RRM      NLCVKNI----P-KETK---ENE---LLEIFQ---PF----G-LIESIK----LKVNKNVGPYA(1)-YAHVLFSTPEEAKRCLK---QMNGKIL---NGR-ALRID
PF3D7_1454000-RRM      IVHLTNL--VTP-EEVDETLKEE---IEEEAS---KF----G-NLLNIN--IVVDKNLLDALAV----KIYCEYESKDQAQNALN---TFKGRTF---AGR-KVQAS
PF3D7_0205700.1        TIICNNI----P-IHIN---RFE---ITEIFS---KY----G-PLLGQG----IYFG-KK--NS(1)-FFFVKYVHLKDAIKAYE---NLKNEKE---CEH-DFKLS
PF3D7_0205700.2-RRM    TIICNNI----P-IHIN---RFE---ITEIFS---KY----G-PLLGQG----IYFG-KK--NS(1)-FFFVKYVHLKDAIKAYE---NLKNEKE---CEH-DFKLS
PF3D7_0517700-RRM      KLFIRHL----S-AKIS---DSS---LLNMKI---HM----P-----------TDEE-NK--TK(1)-ICFVTFNDSFQANEAVK---ILNKLKL---DAK-HILT-
PF3D7_0610200-RRM      VMYIGNI----D-KYIE---DND---MVKMLE---IF----G-NVIKWQ(1)-QRNP-STNELM(1)-FGFCEFSDIYEVYLCMN---ILDNIKL---GDK-HLKVN
PF3D7_1110400-RRM      EIYIRNL----H-IDIT---KEE---IYNLFD---GY------RIKRIN(1)-LNKK-GK--TA(1)--AYVEFDNIEDMNRSLE---LNGKMIY---SGN-NSFEH
PF3D7_1235300-RRM      T-LIVNV----P-PNTT---RKD---LMNVFS---QF----G-NVDLTM(7)RHPNK-EWTATS(1)-YSFVRFSTNIEARKTLT---AATCGLI---KIR-GSKVR
PF3D7_0603100-RRM      MLILKNM----D-GNIL---IKD(4)LNVTFD---KN------DVSCIY----LFND-IKGSSK(3)-FCFIEFYNINMAKKVMN---NMEKNYYLNFQDN-YLKLD
PF3D7_1132100-RRM      T-LIVNV----P-PNTT---RKD---LMNVFS---QF----G-NVDLTM(7)RHPNK-EWTATS(1)-YSFVRFSTNIEARKTLT---AATCGLI---KIR-GSKVR
PF3D7_1353400-RRM      MLILKNM----D-GNIL---IKD(4)LNVTFD---KN------DVSCIY----LFND-IKGSSK(3)-FCFIEFYNINMAKKVMN---NMEKNYYLNFQDN-YLKLD
PF3D7_1131000-RRM      SIYIYNL----T-RNVS---INH---LKEIFM---NF----G-TLKDVQ(52)EEDNDNNDDNN(19)CVHIKYENSKEAQAAKE---FMDGGQI---DGK-IISVK
Consensus/70%          plbl.Nl....s..phs...ppp...lbpbFp...pb....G.pl.phb....h.pp..p...p....btalpaps.pptbpAbp....bss.bb...psp..lplp
```

Key for residue coloring in alignment and consensus sequence

Description	Residues	Marker	Bold	Italic
Negative	DE	.	b	i
Aliphatic	ILV	l		
Positive	WKR	+		
Tiny	AGS	t	b	
Aromatic	FHWY	a	b	
Charged	DEHKR	c	b	i
Small	ACDGNPSTV	s	b	
Polar	CDEHKNQRST	p	b	
Big	EFIKLMQRWY	b		
Hydrophobic	ACFGHIKLMRTVWY	h	b	
