# Supplementary figures and images for: A bioinformatic survey of RNA-binding proteins in Plasmodium
Source: BMC Genomics. 2015 Nov 2;16:890. doi: 10.1186/s12864-015-2092-1 (PMC4630921; doi:10.1186/s12864-015-2092-1)

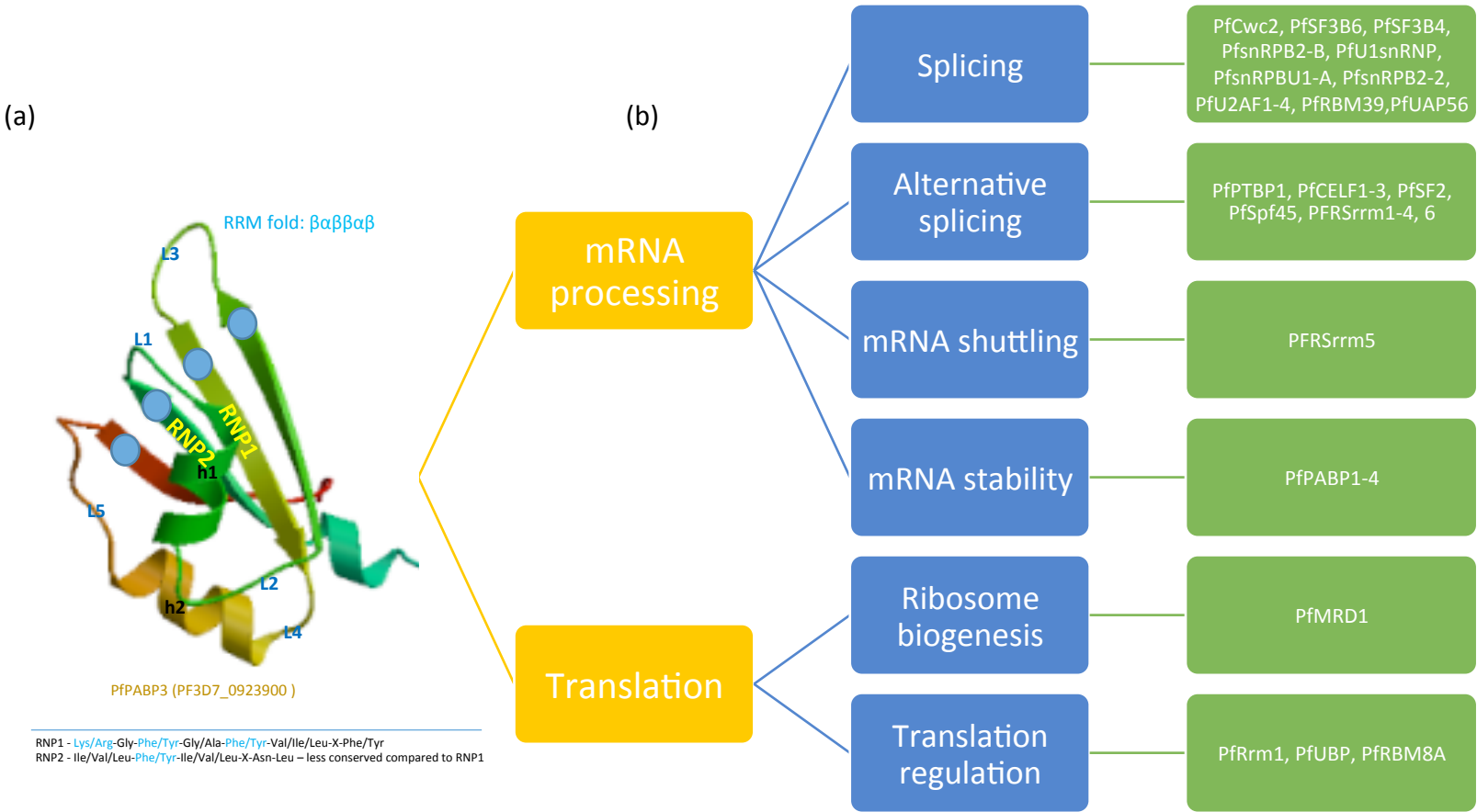

Supplement: Additional file 3: — An expanded functional description and 3D model of the RRM domain in Plasmodium. (a) A representative 3D model of a RRM domain constructed using PfPABP3 (PF3D7_0923900) as a query and PDBID: 2jwn as a template using default parameters as described in the Materials and Methods section. The canonical RRM fold is marked on the 3D model, where L-stands for link; h-helix, 1–4 β-sheet, and RNP1&2 are conserved features of RRM domain. (b) A categorization of putative functional roles of RRM motif in P. falciparum and their associated genes. (PDF 75 kb) [file 12864_2015_2092_MOESM3_ESM.pdf]

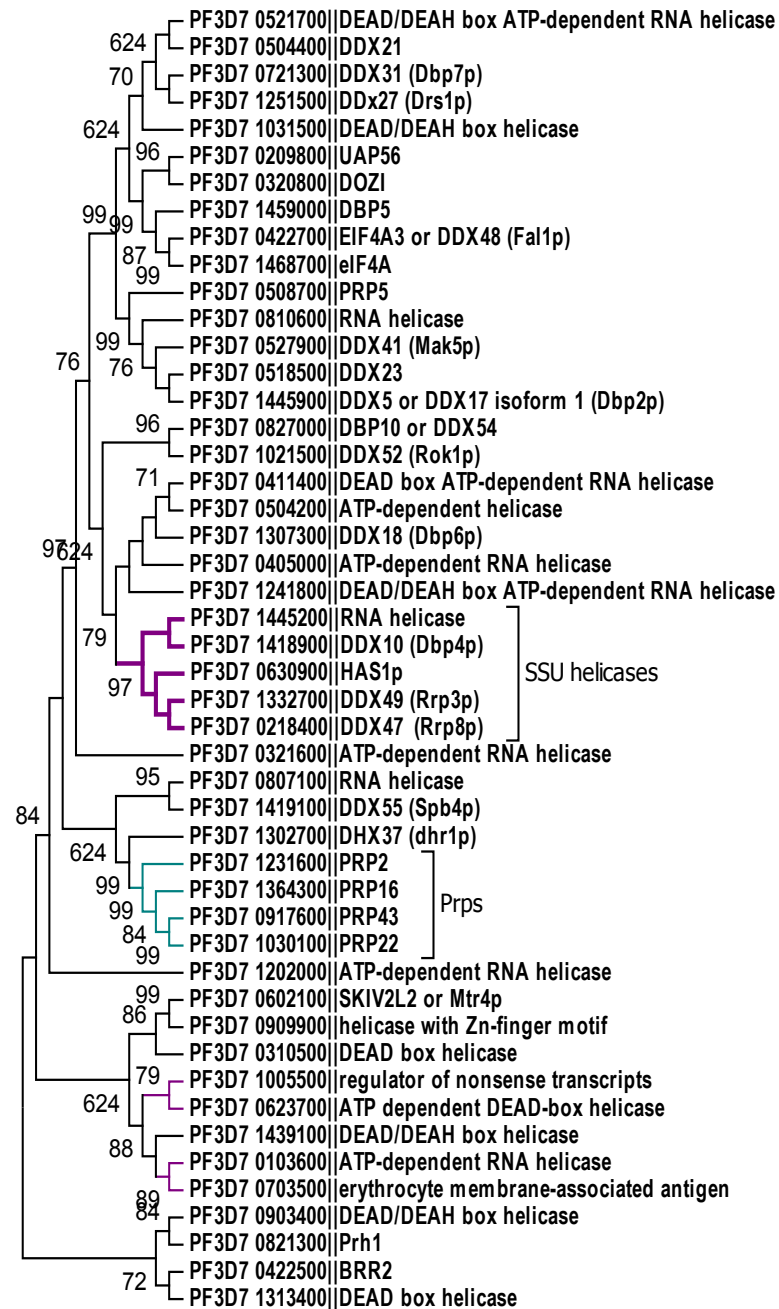

Supplement: Additional file 6: — A phylogenetic representation of the RNA helicases found in P. falciparum. Phylogenetic reconstruction of 48 PfRNA-helicases show uniformly higher support for all the tree branches, which is suggestive of deep-evolutionary conservation at the sequence and probably at the functional level. Two subfamily clusters representing two functions, small subunit rRNA helicases (SSU) and pre-RNA processing (Prp) have monophyletic representation. (PDF 57 kb) [file 12864_2015_2092_MOESM6_ESM.pdf]
