# Supplementary material for: A bioinformatic survey of RNA-binding proteins in Plasmodium
Source: BMC Genomics. 2015 Nov 2;16:890. doi: 10.1186/s12864-015-2092-1 (PMC4630921; doi:10.1186/s12864-015-2092-1)
Supplement: Additional file 4: — A list of Poly(A)-binding proteins retrieved from Plasmodium genomes along with their putative cellular locations. (PDF 63 kb) [file 12864_2015_2092_MOESM4_ESM.pdf]

Additional file 4

**Common name**	**Pf Gene ID**	**PY Gene ID**	**Predicted location of Pf genes**	**Remarks**
*Pf*PABP1	PF3D7_1224300	PY17X_1441700	Pf3D7_06_v3: 1,210,420 - 1,212,762 (+)	PABPC1 poly (A) binding protein, cytoplasmic 1 (A component of *Pf*RNA granules)
*Pf*PABP2	PF3D7_0629400	PY17X_1129700	Pf3D7_09_v3: 966,795 - 968,069 (-)	
*Pf*PABP3	PF3D7_0923900	PY17X_0828100	Pf3D7_12_v3: 988,628 - 991,255 (+)	
*Pf*PABP4	PF3D7_1360900	NA	Pf3D7_13_v3: 2,440,438 - 2,442,909 (-)	
