# Supplementary material for: A bioinformatic survey of RNA-binding proteins in Plasmodium
Source: BMC Genomics. 2015 Nov 2;16:890. doi: 10.1186/s12864-015-2092-1 (PMC4630921; doi:10.1186/s12864-015-2092-1)
Supplement: Additional file 5: — Multiple sequence alignment of DExD/H RNA helicases from P. falciparum. All the conserved motifs representing helicases are mapped to the multiple sequence alignment. For residue color and meaning of consensus letters please see key at the end of the alignment. (PDF 336 kb) [file 12864_2015_2092_MOESM5_ESM.pdf]

Additional file 5.

```
PF3D7_0903400   (288)DSYNND(112)EDNKNYNDVENQ(336)LRRQQKDERRKAIIAKYFYIS(20)NIEKLYDYLRNEE(161)LEQKIVK--LASD(29)TN----LIKSVDLQI(4)KVLNSM(
PF3D7_0602100   (24)DEEQQQE(55)-NHKKENLYKDIN(29)YSDIQNFDKTNKNVNKIRKLSL----QENNNINNNHING(77)EIKEPLIP--ARTY----------KFELDTFQK---KSIECL--
PF3D7_0909900   (58)YNIYKRE(48)-DINNFIYNDNLL(63)EMKQVIYNKEEMLVEKKVLDDN----ENVDMEKIDHPNG(62)-ISNFNKN--DLLL---NYD-----FELDDFQK---RSIKHLNN
PF3D7_1364300   (105)KDKHVN(60)-KQHHNRYSNKTF(145)KKKSDNINNDKYYSNKPIQST----SYSTVVKDETCDF(94)NNNKDKKN--TLKDKEELLK----LKESLPIYK---SKHELLDA
PF3D7_1231600   (92)ELLYSTV(55)-DKSLYNYDEQIL(123)DKHDKHDKHDKHDKHNKKYHS----KYKELNTSYDNNS(96)-IKKRKEK--KMKK---IID----ERKRLPIYS---YRYDILKA
PF3D7_0917600   (21)ECTYSTN(50)-KNVDSNITNNIN(11)ILNNITNVQNDILSKEN---------NNNNNYNNVQPTT(21)-YSQRYLQ--LLEE---KKK----LPAWSAKKN----FLKLL--
PF3D7_1030100   (120)TLKNED(55)-RKKKNSSGGSSK(214)GKNLVNDDMNKEEDNYISLFD----DINEDFNDLKKKN(123)IYDNIPK--DISR(29)WK(20)QRSKLPIYN---LKNDLMKA
PF3D7_0411400   (29)KQNNYVF(54)-DLNKYCIDKFKK(38)THKISDNYMNSNRCISSNYHMC----RKKKKKYNNFREC(69)-FDSYIKL--SLIK---NFN----VKYLTTVQY---VTFPLF(
PF3D7_0827000   (19)KKKINKS(50)-KTNKNISKNKNG(23)NNYSSSCSYDDKKKEKKKFVLY----KKKKNKSNNNSNN(19)-LSENMCR--SIAS---NLK----YNKPTNIQK---LCITKI(
PF3D7_1439100   (338)YFNYVQ(125)DNGDKNFDDDNN(336)MDILDINQIFGRCGRPQYESH(20)NNTVIESNFLKNI(159)LIEVYLRRLQINN(7)NYI----VQNIIRILY(4)ICLNILKN
PF3D7_0518500   (185)DDYNNN(70)ANQKIFANLSNNE(260)QNRLNMSLNKDRENQHQQHQQ(5)-HHNNNNNNNISCN(131)LSNDLLK--AIKK----AK----YEKPTPIQM---QAIPIA(
PF3D7_1031500   (78)DDVNNNN(55)-NKKPKEDKAENI(214)KKRNTYSNNNKTNRSDIYTKE(14)DKNNLYNNGKMEN(117)LCDEELY--KNIC---LKG----YCNMTTIQK---YSIPVI(
PF3D7_0810600   (53)NNFVYNQ(54)-NNNNNNNNNNNS(85)MFDNNNNNNNNNIKRNNNYMNN----NNNNNINNNNGRM(122)LNELLLS--NIKK----VN----YDKTTPIQK---YSLNII(
PF3D7_0508700   (190)KRYHHT(112)SSSENLNKTHEQ(266)DDDDDVFHKLFVEELKKKTEE----DMKRNEKDNMNES(109)LPSKILQ--ILEK----KN----FKKMYNIQM---QTIPAL(
PF3D7_1313400   (21)TKNYKKT(60)-KKKNNGFANNEH(12)QNDENKINYVQGNFDENVKDIK----YLSDMQKNFIYLL(20)-LYYLILK--FFYN----CE----FIFKEDIER---EEYIMKNI
PF3D7_1419100   (24)YIQKYFY(55)-KRNDLFNKKNVN(7)DQIERIYETVENINIEKKNAYRD---VNVNIDIRNFLLYT(40)-IKKDEINIQKNNN---QEE----IIRKNDLQN---ITFATT--
PF3D7_1241800   (5)IKDMMNDE(46)-DDEDNDEDEDND(11)DDDHNDVKKSSKNKKKNNEEMF----DRSNHFDNFDNIM-----LDVRLRK--ALLY---LFK----YQHPTIIQK---MSICKI(
PF3D7_0521700   (40)DLCACAE(55)-KNKKSSYIKTIN(21)SNNFLFEEVKVDCEITNGLYAY----EIEILSKSYVNVG(67)-YKYNDVA--FFPY---IWG----KYFHIKLHF---ENLK----
PF3D7_1202000   (32)FTTYYNF(45)-KKDEKEYGSKYE(8)EEKKDEEKKYRYDEKIHKGLYSE----DNSTSMNNRYNNN(23)-IDEEIIN--NLKI---ILN----IEKLYMYQY---IIYNEI(
PF3D7_0405000   ---MKRSFFIN(55)-DIMKKEIKKEKK(8)IRNTLSSHCRIVEINDDEEISIL--------DWNNIKSTK-----INEYIIN--SLIN---KFH----FNNFLACQS---NVLEYL(2
PF3D7_0721300   (22)SKGFIKN(55)-MNDDYNNNNIKG(11)VDDDDYDDDDDDNFDENKNCND----NCSSKHKRNVPSK(37)-LSESLIN--TLEK----NE----FIKMTSIQK---MSIPLF(
PF3D7_0504200   (17)IKSYNIN(28)-GKGIILLKDSKE-----GNSLNFKVEDFLCESEKKEKN----KNKESHGSFFFQT(8)--IHKLLIE--NLKN----HN----INTPSVIQY---DLLKYY--
PF3D7_1307300   (28)KLNSHVK(48)-KLKNDGLLNSTP(6)NIIKEDLLYRKNNEKENEEVIKS----NIDENKNVHMNDQ(18)-IDKDVKK--SLLE---IFQ----YKEFTDVQR---IVYDNI(
PF3D7_1445200   (30)EEAEGNN(55)-SREDKKIKIEES(36)NMQINTTDNNLKKEKDDEEEEE----REQEREQEREQEN(67)-ILHSVMK--SLFD----NA----FFKPTEIQS---KTLEKS(
PF3D7_0321600   (32)EEIDTLE(55)-KNDSVEYKIYDN(42)YMEKIYNHNDMKSCDRKEDFKE----YDIDDDDNKFLEQ(77)-IDNDILQ--RIYD----SY----LSTLFPVQS---MVIPIF(
PF3D7_1418900   (5)GNVGKGKR(55)-EKINNDLEKNEN(7)DKTNNNTYDEDELKNNKNKKYKN----NLTIIDKNVLTSA(6)--ISKRTLR--ALNE----NN----FIYMTNIQY---VSLPIV(
PF3D7_1021500   (19)GDLMSLK(54)-EINVEYENINRN(29)MNNINKLNHNMKPYNSDSDNQN----NKEKDMEKNKIDT(33)---EKLLS--TIKN---TLY----FKKPTCIQK---MCIPSI(
PF3D7_1332700   -----MIICAR(30)-KKKRYKYKEREN-----------VDGTRLYNEEQINSY----SDQSNNITFEELG-----VEDWLIK---ISK---SVH----ILYPTKIQQ---LCLPLI(
PF3D7_0630900   (23)KNSIIKK(55)-ENNENNENDEDN(11)TSSEEICDHDKNDKTYTDEIKS----NNSDHNNNNSKEH(11)-ICDALKK--GLKE----LN----FVTLTEIQA---KCIPHF(
PF3D7_0218400   (23)KVFNNEE(50)-NNSDNSFHNSDN(6)NYTNNSDYNIINNNHDNINFIHG----NKNKNHDNSFHNN(28)-ICEEILE--SIKE----LG----WKKPTEIQR---EILPHA(
PF3D7_0504400   (42)KAIHTKK(51)-ERKKKIKYPEIG---EYNDDVNHNKNNDNNGDDSESHL----NDQGKCKFSDLSE-----INKNIVE--FLEN---KKG----IKYMTKIQS---QSFKPI(
PF3D7_1445900   (5)YNNNSNNN(35)-NNNSNNYGKNLA(10)PFEKNFYKEHEDISKLSTKEVK----EIRDKHKITILEG(14)-FPDYVIK--SLKN----NN----IVAPTPIQI---QGWPIA(
PF3D7_0527900   (29)YEPLSVR(51)-NDNNNNNNNNNN(11)DGFKVDFRKTLLETFHKIRLEK----KNDEIDETEEIRK(78)-FPKAILK--GLRK----KN----IKKPTQIQM---QGLPSI(
PF3D7_1251500   (5)MKKKICQD(15)-DKDINGKDNTNR-----IKNVSDRDEDDVEEMKEGTSE----RTSISRNNKNSEN(19)-ISRPFLK--VLYE----QK----FSNPTYIQR---DVIPLA(
PF3D7_1459000   (46)DVKEPAD(55)-QKNKDDPAILQT(106)SEKVSGEENTNNNNNEEVVKK----KHSVLVEDNDDYE(71)-IDNELIQ--ILTY----LK----FLGPSKIQA---YALPII(
PF3D7_0209800   ------------(15)-EDDENILDSKDV--KGNLGNNILNNNNKGGAMRGSYAT----VHTGGFKDFF---------LKPELLR--AISE----SG----FEHPSEVQQ---ETIPAA(
PF3D7_0320800   -----MSYKTNCTNS-NANTNTLNSSSN(7)IILDEEWKKKILEPLKDLRYKTE----DVTKTKGNEFEDY(1)--LKRELLM--GIFE----KG----YEKPSPIQE---ESIPVA(
PF3D7_0422700   -----------------MEKDDHYKTS-----------------------------------EKYEICTSFENIG-----IDEGLLR--GIYA----YG----FEKPSAIQQ---RGIKPI(
PF3D7_1468700   -------------------------------MSTKEETFNNENDIEG-----------NTEEIVDTFDALG-----LNEKLLR--GIYS----YG----FEKPSAIQQ---RGIKPI(
Consensus/80%   .........p.....pppp.....p........p....p........p.........p.p...ps..........l.p.bbp...l.p..........b.....lQ....b.b..h..
```

```
PF3D7_0903400   )TGKQ(8)ILIYR(10)EHLSEKDV(17)NLFEEYVKLK(28)GKENKKE(110)ILVSCPTSSGKTFICYYVMDKVLRLNND(26)KGYSKYG---GNKLCS(11)LDSQI---IIII
PF3D7_0602100   -ERNES--VLVSA----HTSAGKTVIAE-YAIA----LG----LRDKQR------VIYTSPIK----ALSNQK-YRDLGEEFK----DVGLITG---DISINP-----EASI---IVM
PF3D7_0909900   -KH-----VFVAA----HTSAGKTLIAE-HAIALSIKLQ(2)-------------AIYTSPIK----ALSNQK-YYEFKNIFK----DVGIITG---DVKMNV-----NANC---IIM
PF3D7_1364300   -YNNNI--IIIVG----ETGSGKTT----QIVQ---YLY(2)-GYHRNGI-----ICCTQPRR----VAAVSVAYRVSYEMNV----DIGSLVG---YTIRFEDNTTKDTKI---RYV
PF3D7_1231600   -KNNKI--LILVG----ETGSGKST----QLTQ---YLY(2)-KYHMYGN-----IICTQPRR----IACIAIANRVAEEMNV----KIGKEVG---YVIRFQNKTSEATKI---MYM
PF3D7_0917600   -KKNNV--LIIVG----DTGSGKTT----QISQ---FVL(2)-KYTEKKS-----IAVTQPRR----VAAMSVAARVSEELDV----ELGTYVG---YTIRFEDKSSNKTII---KYL
PF3D7_1030100   -EKNNV--LIVIG----ETGSGKTT----QIPQ---YLH(2)-NYTEKGI-----VGCTQPRR----VAAMSIAKRVSEEFGC----ILGQEVG---YSIRFDDCTSNDTII---KYL
PF3D7_0411400   -KNYD---LLISS----FKSSGKTL(1)-YMNC---LVHKIIIQINNLRK(10)-GLIICPNV----ILVEQS-YGILKKLIM(6)-ICYYMHGKK-YINIQD(6)KKRPHI---IIT
PF3D7_0827000   -HRRD---VICIS----KTGSGKSL(1)-YLCT---LID----ILKEHNKYYGIRGLIILPTK----ELVIQI-YKLCKKICK(5)-NINIIIG---GVSIIK(6)-QNLDI---LLC
PF3D7_1439100   -SNLI(8)AILRRLPI-NCGIFRHF(1)-YKNE---MLE(39)DINEENE(110)IFLLNTLN(26)PLSNQIPPQITVQFLS(92)NLLRCEG(9)KAIVNE(25)ENKEN(6)IIC
PF3D7_0518500   -EMRD---LIGIA----ETGSGKTA(1)-FVLPMLSYVK----QLPPLTY(8)--ALVIAPSR----ELAIQI-YEETNKFASYCSCRTVAVVG---GRNAEA(5)RRGVEI---VIG
PF3D7_1031500   -KKIN---LLACS----QTGSGKTF(1)-FLCP---IIS(2)-KKENEIL(14)-CLILCPTR----ELVIQI-QNEVNSLTKNMSIVCMSFYG---GETMKD(6)EKQGDI---IIC
PF3D7_0810600   -NRND---LIGVA----QTGSGKTA(1)-YLLP---IIN(2)-LINDPPK(22)-CLILAPTR----ELAVQI-FYDAKKFCFETGIKPVVLYG---GNNIKT(6)-KGADI---IVA
PF3D7_0508700   -CGRD---VIAIA----ETGSGKTL(1)-YLFP---VIR(2)-LHQEPLR(6)--SIILTPTR----ELSIQV-KNEAKIYCKAVNIEILAVYG---GSNIAR(6)-KGVEI---LVG
PF3D7_1313400   -IDRN----IYES----KNKSRKYF(5)-RFIPYSEKYS----EISDIKE(19)-VLILCPSK----ELCVQVAQNILSFMNN(92)DNNNNSN---NNSNNN(19)KGVRY---LIG
PF3D7_1419100   -CNSN(4)KQETN----KEKHEQKI(3)-YNEQ---NIQ(2)-NQTKKPH(22)-AIIITPTR----ELCIQI-YNLINKFLF(26)NCLLFRG---AVKISE(12)NRYQI---IIS
PF3D7_1241800   -NGHD---VIISS----KTGSGKTM(1)-YLIP---VVH(5)-NLNEKDH(6)--CIIICPTE----ELCLQI-YDVTKKLCT(3)-DIITVNH---NVNNTF---YEHPTI---LIS
PF3D7_0521700   -YGDR(8)ILLTS----NCNDNDTL(17)FKLEKANMQT(1)-NYQNNNS(21)-CIVLCPTR----DLAQQT-YNNYLTYSE(7)-NIGILVG---GEQRNK(6)DGSYNI---VVG
PF3D7_1202000   -KNSKH--LLIYN----KTGTGKTL(1)-YLLP---LIQ----KLINDKF(22)-ILSIYPTL----NVCILSDEKESYNMNNNINNDIIVNIK---RNNINP(12)YGIHF---YLC
PF3D7_0405000   -YKYG(8)IYIEV----PTGLGKTL(1)-YIIS---IID(2)-LYNNENTLY---CLILTATD----ELVNQI-INVINIFEL----RNLKCM----NIDMNN(17)SDCNI---LVT
PF3D7_0721300   -KPND---IFLKS----MTGSGKTL(1)-YAIP---SIE----KILNMKE(11)-VLVLSPTR----ELAIQI-NNLFCILTK(5)-VASCITG---GEKKKS(6)-KGISI---LTC
PF3D7_0504200   -DEHN(5)IIIAA----ENGIGKTL(1)-YIIF---LIN(3)-KKGTNKNTC---TLILQYNN----LLCNQC-YDLLKKLSK(5)-IVNLKYE---GIINIKNHLI--------VIC
PF3D7_1307300   -KEKKQNDLLIQA----KTGTGKTI(1)-YLLL---SIN(2)-IKNKILSVH---TLIIVPTR----ELANQI-YNECKLLLT(5)-NVLTLTG---GIKRRD(6)RIKPDI---IIC
PF3D7_1445200   -NDKND--IVVIS----KTGTGKTL(1)-FCLP---ILN(2)-LINKLKE(12)-CLILVPTR----ELALQI-LKHFNYINKYINLFISTIIG---GLNLNK(6)-KKPEI---LIC
PF3D7_0321600   -SGRD---HIVTS----NSGTGKTL(1)-YIISLIIHMM(2)-KKIKKNS(25)-ALILTPTK----ELCIQI-YDDINNICC(3)-KTSVLCS---GIDYKK(6)-KGTDI---VIC
PF3D7_1418900   -LNKH---IYAQA----QTGTGKTL(1)-FCIP---LIE(2)-YRNSIDN(6)--GIIITPTR----ELVFQI-FEVLNMLNKYHKLNICCAIG---GKNEEK(5)-SYANI---IVC
PF3D7_1021500   -NDYN---TICIS----QTGSGKTC(1)-FLIPLLIKLN(28)NNNKNNN(22)-SLIIVPTN----ELACQIYEQALLLFESYKHINIIHLNK---LNDIIE---NNVIDV---CVC
PF3D7_1332700   -QGKN---VIGSS----ETGSGKTI(1)-YCWS---ILQ(2)-NKNVYGIF----SLILLPTR----ELVFQI-IEQFHLYGSKIGVMILSCIG---GFSLIE(6)-TKPHI---IVG
PF3D7_0630900   -SGKD---ILGAA----KTGSGKTL(1)-FLVPSINILY(2)-KFLPKNGTG---VLIISPTR----ELCLQI-YQVCKDLCKYIPQTNGIIIG---GMSRNE(6)-HGINI---LIA
PF3D7_0218400   -LKKD---IIGLS----ETGSGKTA(1)-FIIP---ILQ(2)-KVNKQSFY----ALVISPTR----ELCIQI-SQNFQALGMNLLINICTIYG---GVDIVT(5)AKKPNV---IVS
PF3D7_0504400   -EGKD---IIGRS----ETGSGKTL(1)-FALP---LVE(28)DDVDNDN(21)-ILVLEPTR----ELSKQV-ENTFKEISQFYNFNIMSIYG---GESYTY(6)-KGIDI---LTG
PF3D7_1445900   -SGKD---MIGKA----ETGSGKTL(1)-FILP---AFV(2)-LAQPNLK(6)--VLVLAPTR----ELAEQI-RQECIKFSTESKIRNTCAYG---GVPKSG(6)-QGVHI---LIA
PF3D7_0527900   -LGRD---IIGIA----FTGSGKTI(1)-FVLP---LIM----KCLEAEI(11)-GLIICPSR----ELATQT-HNIIKYFCE(10)RSLCMIG---GISAYE(6)-KGIHM---IVA
PF3D7_1251500   -EGKS---ILANS----ETGSGKTL(1)-FVLP---ILERLLQSVNIKMR(12)-ALILLPTR----ELSLQC-YDVIRSLTKYVTITYSLFCG---GIDIKQ(6)-KRNDI---FVC
PF3D7_1459000   -SSNKN--LIAQS----QNGSGKTL(1)-FVIA---MLC----KINRTLSSLQ--AVCICPTR----ELSQQN-YDVVCNFTKYLNVKVFLAVP---LCERYN--KSGGYQI---YVG
PF3D7_0209800   -TGTD---ILCQA----KSGMGKTA(1)-FVLS---ILQ(2)-DTNENQD(21)-CLGLAHTR----ELAYQI-KNEFDRFSK(5)-RCEVVYG---GISMNK(7)DNIPHI---IIG
PF3D7_0320800   -AGKN---ILARA----KNGTGKTA(1)-FAIP---LLE----KCNTHKNFIQ--GLILVPTR----ELALQT-SAMIKELGKHMKVQCMVTTG---GTSLRE(5)YNVVHI---LCG
PF3D7_0422700   -NGRD---VILQS----QSGTGKTC(1)-FAVG---ALN----CVNRNLNETQ--VIILSPTR----ELAEQT-QKVCLALADYIHVTIYCCIG---GKKMSD(6)-NGVHV---ISG
PF3D7_1468700   -NGYD---TIGQA----QSGTGKTA(1)-FVIS---SLQ----LINYDYVACQ--ALILAPTR----ELAQQI-QKVVLALGDYLKVKCHACVG---GTVVRE(6)-QGVHM---VVG
Consensus/80%   ...pp...llh.t....psGsGKTh....ahh.....l.................slhl.Psc.....Ls.Qh.bp.h..b......ph..hht...h..b.p.......pl...bhs
```

```
PF3D7_0903400   SILENIL(12)MNVSKFISKIEYIIFDEIH----(4)KEFYGSQ-I---ENII--HLINCPF(84)EIIIILFEL(7)HLVE(9)YTRCI(36)LLSNYMKNSFYIKDENEEDI(49)
PF3D7_0602100   EILRSML----YRGSSLTKEVKWVIFDEIH---Y(4)DRGVIWE------ETI--ILLPLMV----RFI--FLSA----TIP(8)VSSIK(18)YIYPTSSESVFLICDENKDF(27)
PF3D7_0909900   EILRNLL----YLNDNIINNIHCVIFDEVH---Y(4)DRGVIW------EESI--IMLPHHV----QIL--LLSA----TVP(8)VGFTK(34)KFYSSAFKEIYVKIREKQEA(43)
PF3D7_1364300   GIL-----LRETLNDKELDKYSVIIMDEAH---E----RSINTDVL---LGIL--KNICLKRNDL-KLI--VTSA----TID---AKKFS----AFFGN--APIYNIQGRTFKV(35)
PF3D7_1231600   GMFLRLL-----LYNPTLEDISVLIIDEAH---E----RALHTDVI---LPIV--KDICNFRENI-RVV--ISSA----TLD---AEKIS----TYFNC--APIFYVPGRKYNV(49)
PF3D7_0917600   GML-----LRESMSDPLLTKYNTIILDEAH---E----RTLATD-I--LFGVI--KNIQEKRNDL-KLI--VMSA----TLD---AEKFQ----KFFNN--SKILNIPGRLFP-----
PF3D7_1030100   GML-----LRETLSDTLLTKYSFIILDEAH---E----RTISTD-I--LFCLL--KDVVRKRADF-KLI--VTSA----TLD---AEKFS----TYFFN--SPIFTIPGKIFP-----
PF3D7_0411400   GYLMNFI-KYFKNFSQIFFLCDTLIIDEAH---F(3)-NNYMKN-I---LIIK--NILPKSH(85)RLI--NFNL(10)IYP(9)IGKLW(32)NKIIIKKNKIIIKKNNIIIK(52)
PF3D7_0827000   GRLSFLL-QETKLS---LEKVEILIIDEAD---R(3)-LNYYND-M---NVIY--KSLNNTSK---QTI--LVSA----TLP-TNVENYF----KLKLNNPDVLFLTSDNTINEKVH-
PF3D7_1439100   EK-LDVI-TRNWKNKKFVKNINLIIFDEIHLLGQ(14)KNMQNE-L(4)RLIGLTTVITSVD(58)KNVLIFVSS(10)IIS(9)FLHVE(36)AENINTFEYTEYKMNINRKD(48)
PF3D7_0518500   GRLQDCL-EKAYTV---LNQCNYVILDEAD---R(3)-MGFEDT-V---HYIL--DKIPTSN(22)YRLTQMFSA----TMP-PSVERLS----RKYLR--APAYISIGDPGAGKRS-
PF3D7_1031500   GRLLDLL-NSCKVS---LSFIKYLIFDEAD---E(3)-LGLKKQ-M---DEIIFQKDLSPKDTR--QTI--FFTA----TFS-DKLKEHIE---KYMST--PYVYLNIKQKRET(5)-
PF3D7_0810600   GRLNDIL-EKGKIK---LFLTTFLVLDEAD---R(3)-MGFSPQ-I---RSIV--NDYDMPG(24)QTI--MFSA----TFR-KEIQVLA----KEYLC--KYTFLLVGKVGSTNEY-
PF3D7_0508700   GRIIDIL-TISNCKVTNLNRVSFVVLDEAD---R(3)-LGFESQ-I---YNIL--RNCRKDK----QTA--MISA----TFP-NYIQNMA----KKLLY--KPIEIIVGEKGKTNN--
PF3D7_1313400   TCFRSYL(4)KENLKNFLQSIKYIFYDEID---K(14)KNIKSK-T(4)LETL--MYINKKN(15)RKIFKLLCL----NKN-NAKKKIY(17)TRIDNITNLIDIPIKNNEKK(48)
PF3D7_1419100   GKLSSLL(5)KYFN---TNELKYFVLDEGD---K(3)-DSYIRY-M---ENII--KNIQAYD----YTT--CICS----ATC-LQEENVYN---LFQVK--KKNFIKCINTNNQ(14)
PF3D7_1241800   KDLCTHI(4)KKNNLDILMNLKILILDEAD----VLHTQEFQSY-L---KTLT--SYLPKKFNKKYQIV--MASA----TLK-RNILEKT----KLFLH--NPIYVSHEQKNES(42)
PF3D7_0521700   FKLIECI-RKNSIK---VNDIRLLILDEAD---E(4)DEKTVLE-I---KDSC--MKYGHRV----QTL--FFSA----TLQDKNVKDCI(26)LYYVNDENSNLSYIIKENDN(43)
PF3D7_1202000   NKLEYFI(11)NVMNNILNNINTIVIDEFD---Y(4)RKNILNFFF--KKDLF--NEIHNNN(18)NIY--LFTA----NIN-EVIKKKI(10)DFINGIKEHIQKQVAEQHNF(48)
PF3D7_0405000   KKFEGLF----YSNEDIFMNLRFLIIDEVD---K(4)TQCNICS-I(4)TNLV--RKHQNNC(10)QKI--LVSA----TLC-KVSDNLM----SLDLY--RPIFFYYMLNYKR----
PF3D7_0721300   GRLLDHL-ENTKSLK--LTFLKMVILDEADKIIY(14)RKIKQEEF---SKVH--KKKKKEE(34)QMI--FISA----TLN-HAMKTLA(36)SCMNRENSPLNIHNNDDNDD(42)
PF3D7_0504200   VRLISYI(4)ENVFSTFFENLDFLIMDEVD---I(3)-NPYIRN-M---KIIF--DELNKLKNE--KSVSIITSS----TLC-NKGKKSIYN--NVIKYITNPIVIKTNYFHNIH---
PF3D7_1307300   GRLLDHF-ESTYLFNTLFENLKMLIIDEAD---Q(3)-TGYEND-I---HRLL--TYLPSNR----RNI--LLSA----TLG-YNLDEIRK---KMCKS--DYIYLNCVKDISKHTSD
PF3D7_1445200   GRLKYFL(4)PIKYIYQMKNIRYLVCDEID---K(3)-ISFMKD-I(4)KHIY--KSVGDKKKKLIQTF--LLSA----TLS(9)MTKLLN---SIIIRKDKSFIINLSNEQNV(10)
PF3D7_0321600   KTLIHFV-NKKYFS---LVKIKYVIVDEFD---L(3)-KQFVHL-V---TSVL--KNIRT------DSIKGMFSS----MVS-EPMYGLVK---TYLNK--KYITLKIENKYSM----
PF3D7_1418900   GRLLYHL-ENNYYCN--LDYLSTLIIDEID---K(3)-KSFYDN-L---KNIL--LYKPKENC---QIC--LFSA----TIC-KFLNVILN---TFHIKDYEYVSINDNDKYIESNN-
PF3D7_1021500   MILLNMI(15)KVS---LKKCFFIVFDEVD---K(3)-IKFLEQ-V---NTLL--KEIQNRKI---QKI--FTTA----TLP-GNIKNFIS---TLCP---NYTVVYFGKNINTINNN
PF3D7_1332700   GRISDIL-ESSIDIQNCFKRLRFLVLDEAD---L(3)-KCFEDK-L---QNIL--NNLPKNYANERKTL--FFSS----TIT-NSLQLLID---TFPYN--NLILVNVNKKQKPPKN-
PF3D7_0630900   GRLLDHM-QNTKEF--IYKNLICLIIDEAD---R(3)-IGFEEE-I---NLII--KRLPKKR----QTA--LFSA----TQT-TKVESLI----RLSLQ--KPIFIEVTTKIATVER-
PF3D7_0218400   GRILDHL-NNTKGFN--LKNLKYLVFDEAD---K(3)-QDFESS-I---NKLL--LILPPNR----ITF--LFSA----TMT-KNVAKLK----KACLK--NPVKVEVSNKYSTVST-
PF3D7_0504400   GRIIDHI-EKKNLS---LQNIKYVVLDEAD---E(3)-LGFTHD-I---ERIL--SNINLKEA---QVL--LYSA----TTP-SWIKDIS----SKYLK--NPFCIDVVDSSNK(7)-
PF3D7_1445900   GRLIDLL-EQNVTN---LMRVTYLVLDEAD---K(3)-MGFELQ-I---RKIV--DQIRPDR----QTL--MWSA----TWP-KEVQALAK---DLCKE--QPIQVNVGSLTLTACR-
PF3D7_0527900   GRLNDML-NKKRMT---LEQCRYLCFDEAD---R(3)-LGFEEE-V---RNTL--DHFSNQR----QTL--LFSA----TMP-KKIQEFA----KSTLV--NPIIINVGRAGAANLD-
PF3D7_1251500   GRILDLL---LNSSSDFINYLEIVVFDEAD---K(3)-LGFKEE-C---LKIL--DVCKFKK----QIL--FFSA----TLT-SDIKQLA----NFSLK--NPVFIQSGMSFDK(49)
PF3D7_1459000   GKTLDFL-KRKFID---TKNIKLFVLDEAD---D(4)KNNMSSQ-V---ETIK--RFLPRSC----QIL--LFSA----TYN-DSVRKFA----DQFAPKATKISVRQEDLTLKC---
PF3D7_0209800   GRILALI-REKYLI---TDKIQHFVLDECD---KCLEKLDMRSD-V---QKIF--ISTPLKK----QVM--FFSA----TMA-KEMRDVC----KKFLQ--NPVEIFIDDEAKLKLHG
PF3D7_0320800   GRILDLA-NKDVAN---LSGCHIMVMDEAD---K(3)-PEFQPI-V---EELM--KFLPKEK----QIL--MYSA----TFP-VTVKEFR----AIYLS--DAHEINLMDELTLKG--
PF3D7_0422700   GRIYHML-NLRHLK---CKYIKQLVIDEAD---E(3)-KGFKEQ-V---YDIY--RFLSPNT----QII--LSSA----TLP-QEVLEIT----NKFMH--KPVKILVKRDELTLEG-
PF3D7_1468700   GRVYDMI-DKRHLG---VDRLKLFILDEAD---E(3)-RGFKAQ-I---YEVF--KKLVPDI----QVA--LFSA----TMP-QEILELT----TRFMR--DPKTILVKKDELTLEG-
Consensus/80%   .bl..bl.........hp.hphllbDEhc...b....bsb.pp.h.....lb..p.b.......p.h..bhSt....Tbs...h.pb.....pb.....p.h.h...p........
```

```
PF3D7_0903400  RDNVPKEKLFQEL(9)-YISNRTND-LVK(30)PCIV---FNFERKELE----DMTINLIN(91)VY(22)YKSI-LIEG(5)GLH(5)YKFT--IIVESLF-R----LGFVKIIFSN
PF3D7_0602100  KYTKKMRKTTYDIE---KIVQMCHSRNYT----PLII---FAFSKKECE----VNATAMHK(16)EN(22)LRGIGIHHG---GLL---PIIK--EIIEIMF-Q----ESLLKVLFST
PF3D7_0909900  NNINNNNNNNNNVI---GYYEYCKQ-KRK(30)PVVL---FCFSRIKCE----TYAKCMPH(16)KE(22)EKGIGVHHS---GLL---PILK--EIVEILF-S----KGLIKVLFAT
PF3D7_1364300  LIFMTGQEDINAT(6)-RFYEVYES-YKE(12)KNILNEDNNNNNNDSN----IKKKVDGD----NN(8)-FYIF-PIYS---QLS---SEQQ--SKIFKKY-D------LRKIIVST
PF3D7_1231600  KLNELAPKYRNLV(11)YQARIFED-VTE(30)ENEK---NDKNEKNDK----NDKNYKNE----KN(22)SKNKRNINE---EIE---ELKR--INTNNKI--------RRKIILST
PF3D7_0917600  IFYTLQAEKDYVKV---VIRTVYDI-HINEEEGDILV---FLTGEEEIE----MTKKEIER----VV(8)-LVVL-PLYS---SLP---PAQQ--QKIFEPPPK(5)DKNGRKCILAT
PF3D7_1030100  ILHSKEPESDYVEA---SLITVLNI-HLNEHPGDILV---FLTGQDEIN----TACEILHE----RM(10)LIIL-PIYS---SLP---SEMQ--SVIFEPA-P----PGCRKCILAT
PF3D7_0411400  YHCSHHNKNDNKYI---PTHVLLKQEYLI(24)NIVI---FMPTVKILQ----FFYVIYKH----YIFKG-YIFLLYLKL---KKI(5)QDIKSYLANHSTYYD----KDTVNFTVSS
PF3D7_0827000  FLFCRSYEKYALLL---RLILIFKKKKLG----KTMI---FFCTKYHIL----FFSNILKH----FK----IHHS-ILYG---NSD---TSFR--FEQINNFTK----NEHIQFLLVT
PF3D7_1439100  KEYKQYNNEDDNK(5)-YNNNFCDNEIYD----YNLL---FNNSKLSEE(8)-LFQNYLNL----IE(8)-KYGIGIHHA---GLN---ENDK--TIVEYLF-L----NKIIQILICT
PF3D7_0518500  QKLEFLTEGKKKQ----KLQEILEM-YEP----PIIV---FVNQKKVAD----IISKSITK----MK----YKAV-ALHG---GKA---QEIR--EQTLSAF-K----NAEFDILVAT
PF3D7_1031500  VKYVPAKSKFIELL---KDIKILKG--------QAII---FVELRHSIN----NVFNFLKT----KG----YNVD-YLHG---KMS---QIRR--QSVFENF-R----KKSVQILIAT
PF3D7_0810600  QNLVFVEEENKCNY---LLNLLAEN-NNG----LTIL---FVETKRKAD----IIERFLSN----QK----LNAV-CIHG---DKS---QDER--ERALKLF-K----RGIKNILVAT
PF3D7_0508700  YQFVEIIEESKKVF---RLLKLLGE-WIK--YGLVLI---FVNKQIEAD----LLYLELYK----YD----YNLL-VLHG---GQD---QTDR--QFTLEKF-K----KEENKVLIAT
PF3D7_1313400  QKYIIKVKLPSTIY---HFYHLMTDETYE(16)KILI---LIKNGYSLI----QLKKYLEM----NN----IFAV-LLHE---KLQ(5)NNDN--INNMCEHYE(16)INKYPIIISS
PF3D7_1419100  QNYYIILRNIDKAL---FLFKFLNT-MVN(3)-TVIV---FFPTCLCVE----FFFHLFKN(91)KN(1)-FNFL-KIHR---KMK---DKKR--VATYNKIIN(16)NSERKIIFCT
PF3D7_1241800  AFYYVYKEELIKYI---YLYNLIKIKIIP---YKSII---FTTTIHDAY(10)VSSSILNP(91)DE(22)DNDDQTLNE---PLS---DNNSCRTYNSDDENE----KTDDTTKLNE
PF3D7_0521700  KEQISLNIKMNKLK---KLVQIINVFNMQ----NGII---FCRTNLDCD----NVYNFLNA----VG(16)YSCV-ILKG---KMS---NDER--KNNLQAF-K----KGEVRFLICT
PF3D7_1202000  EFFFHKQKISQKVYND-MVYDEYDKLNYR(29)KCII---FANTKEEVE----KLYEISLL----------KPHAVMMHS---ELL---TIQK--NENINLF-K----VGKKNVLITT
PF3D7_0405000  EFYFFTKNKYTKMY---TLIKLLLD-DIP(4)LSMLI---FCGDEDSSH----TLYRYLTI----YF(7)-YSIK-EYSR---ELS---NKRK--KKILTNF-L----NQRVHILICN
PF3D7_0721300  KQYCILIDMKQKF(28)YLQILLKNIYWP(35)QNILNNNYYNNNYNVG---NISYKNINL(8)-LN(22)VNIY-ILHG---NLS---KEDR--LGNFMDF-S----KTNNSILLCT
PF3D7_0504200  INYHFIKASNYYI(5)-IIKHILSKQNYK----KVLI---FCNTLKSSN----TAFSLLKLH---------FDNIFLFNS---TVT---KEDQ--TIILNHF-K----NSQNPILVTT
PF3D7_1307300  IQYVLFHKAIDTTI---ILYNLLIE-HMR(5)YKIIV---FFPTARATS----FYAQFFINQ---LK----ISVY-EIHR---KKE---MAHR--QITANRF-S----MESVGILFTS
PF3D7_1445200  TLYIVKLNERDIVC---KLFYLIKS-YFS(30)KIII---FVNTIKSAK----QLNAIFKH----LF(22)VNIY-SIHS---KQK---LKER--LENINKFSQ----QNHKAILFCT
PF3D7_0321600  EKFYILEECSKYNY---LIDNIKKCSDKG----QGFI---FCNSKKNVM----LLYDKLKK----ERSFN-YISFDFIYG---DLL---QNER--IYKYEKL-K----NKKTNILITT
PF3D7_1418900  QIYIECDIYEKINY---LYTFLFSK-KNK----KIIV---FFSTCKQVR----FMYEVFKK----IKVGV-MKFL-QLHG---KLK---QTSR--LNTYHFFSK----KKNFVCLFTT
PF3D7_1021500  QELLYVNNEEEKLL---VLNNLIKNKEIH---IPVLI---FVDSIIKAN----MIYTNLHKS---------VSYIALLTS---EKS---KEER--KIIFQKF-Q----QGHIWYLICT
PF3D7_1332700  QRYIYVEEIAHIT----YLIYILKN-KVN--NLSGII---FTANSYKCE----LVYTVLNM----LG----IDNVDAMHS---SKD---QKNR--FATLAKF-K----NGLCKILVAT
PF3D7_0630900  QGYALVDEDKRFLL---LFTFLKKN-MSK----KIMV---FFNNCMSVQ----FYNDLLNY----ID----IPTY-CIHG---KKK---QNKR--LKSFHDF-S----AAKCAILLCT
PF3D7_0218400  ETYIFLPLKYKYT----YLSSLCFHYQTR----NIII---FTNTCATAQ----KLNFFCRN----LG----LKSI-CLHG---KLT---QNQR--LSSLNSF-K----VNKYNILIST
PF3D7_0504400  IKTPYDIKEKALLL---EDIILVKS-NGG----QVII---FTRTKLEAD----ILCSEGSF----KS----LSFA-VLHG---NIA---QSTR--EYTMQRF-R----QGMFQILIAT
PF3D7_1445900  KQEIYLLEEHEKIG---NLKSLLQR-IFK(3)-RIIV---FVETKKNAD----FITKALRL----DG----MPAL-CIHG---DKK---QEER--RWVLNEF-K----TGKSPIMIAT
PF3D7_0527900  QEVEYVKEEFKLS----YLLEVLQK-TGP----PVLI---FCENKKDVD----DVHEYLLL----KG----VNAV-AIHG---NLG---QSER--QEAINLF-R----EGKKDILVGT
PF3D7_1251500  QEFVNIIQEKYRKA---SLLYLCNNIYKN----HCII---FFKTKRETH----LMYLLFDL----LN----LRCA-ELHG---SMS---QKKR--IESIMKF-K----KAEVDFLLTT
PF3D7_1459000  QYYLITENDEQKYY---YLSELYCSMTIS----QCVI---FVNSKKSAY----NLYNFMTE----NS(22)ANVL--------GMD---PKTR--DTLMADF-K----KGISKVLICT
PF3D7_0209800  QHYVKLQEKDKTR----KLIEILDALEFN----QVII---FVKSVTRAI----TLDKLLTE----CN----FPSI-AIHG---GLE---QQER--IERYDKF-K----KFENRILVST
PF3D7_0320800  QYYAFVKERQKVHC---LNTLFAKL-QIN----QAII---FCNSITRVE----LLAKKITE----LG----YSSF-YIHA---RMS---QTHR--NRVFHDF-R----NGACRCLVSS
PF3D7_0422700  QFFVSIEKEQWKYE---TLADLYESLTIT----QAVV---FCNTQMKVD----WLTKKMLE----SN----FTVC-KMHA---GMS---QSER--DDIMLKF-R----QCKFRVLIST
PF3D7_1468700  QFYVAVEKEEWKLD---TLCDLYETLTIT----QSII---YCNTRKKVD----ILTQEMHN----RL----FTVS-CMHG---DMD---QKDR--DLIMREF-R----SGSTRVLVTT
Consensus/80%  bbb....p..........b..lhp.........hll...Fh.s.bphp.....h.p.b...........b..h..bp.....b....ppp+.....b..a.p.........hllsT
```

```
PF3D7_0903400  --NLSLGINIPC-RSIIFAG--HTIE---LNSLMF----KQTSGRAGRRGFDLYGNI(307)NTFLS(5)-SLNRI(138)ENADYFKFYKSKLRDYK(147)
PF3D7_0602100  --TFSMGINMPA-KTVVFT(4)FDGL(4)ITSGEY----IQMAGRAGRRGLDDRGIV(269)NNLKQ(8)-FVECL(129)LNNKNLNKYYQMYSKYV(261)
PF3D7_0909900  --TFAMGINMPT-KSVVFT(4)HDHL(4)LTSSEY----TQMSGRAGRRSSDKYGYV(255)ENYKN(13)IYHSS(114)DCLDDIENIERNINAKS(200)
PF3D7_1364300  --IAETSLTLDG-IKYVID---TGYC----KLKVY(19)NQRSGRAGR---TGAGIC(133)EKFTV(6)-TLLNI(18)-IQYKALNKAKEVYSQLI(105)
PF3D7_1231600  --ICETSITIDN-IVYVID---SGLC----KQKIY(19)NQRTGRAGR---KRDGKC(135)MFIIE(6)-LFLNI(29)-VKTQLLSICEKIDIPIT(102)
PF3D7_0917600  --IAETSITIDG-IVYVID---PGFS----KQKVY(19)EQRAGRAGR---TKPGKC(133)MRFSH(6)-TLLNV(26)-RAMTSAQNVRQQLLRIM(121)
PF3D7_1030100  --IAEASLTIDG-IFFVID---PGFC----KIKKY(19)KQRAGRAGR---TGPGKC(133)NKFIM(6)-TYLNI(21)-RALKRAQDVRKQMLSIF(137)
PF3D7_0411400  YIITNDSTIYQN-FNVIHS---YDHS(6)IKNDEF(9)-IKRNGEVTI---KRNGEV(218)YDMGT(8)-DIKHK(65)-IEWHKYKHLLSSCELMY(151)
PF3D7_0827000  --VASRGINITS-VQNVIN---YNLP---FSPKLF----IHRIGRACRTDISGYGIS(301)INYIS(8)-SNENI(139)NINDPSEHYMENKKKLS(306)
PF3D7_1439100  --TLAWGINLPA-YLVIIK(4)YDA-----KTKKY(8)-LQMIGRAGRPQFDDKALA(236)TDFCT(6)-EFYDI(124)YKLKQLDIYNINQLIKF(179)
PF3D7_0518500  --VAGRGIDVHG-VKLVIN---FDMP---KDIESY----THRIGRTGR-A-GMKGLA-----ISFIT(5)-LFYDLKQ---FLISSNNIVPLELANNP(22)-
PF3D7_1031500  --IAARGLDFPD-LELVIN---YDLP---SEFEQY----MHRIGRTGR-I-GKGGMA-----INYFNS---SNKNIID---KLIDHLRKYDQPVPNWLLHFRK
PF3D7_0810600  --VAARGLDISN-IKHVIN---FDLP---SNIDDY----IHRIGRTGR-A-GNIGIA(44)-RNYKY(8)-FNNRF(26)-NNNNPFNNNKGPFSNYN(91)-
PF3D7_0508700  --VMARGIDIKN-IILVIN---YQCP---DHIEDY----IHRIGRTGR-S-NNIGYA(138)SNIYP(8)-NMFED(55)-MKTYNFLSLQENHDKFL(192)
PF3D7_1313400  --DSIRGFHINN-LDMVLL---CNKP---KNVNEY----IHLCGRVGR-R-KKIGYS-----VTLEND-------------KNINIMNNWFNNIKVYF(42)-
PF3D7_1419100  --IISRGINM-D-IHWVIN---YDAA---NKNMTY----IHRSGRTGR-F-DKTGKN(155)LKFIF(8)-HLCYA(20)-INIQSFQIPYKNEEKEK(134)
PF3D7_1241800  DFLYSRGLDFYD-VKCVVN---FDMP---SDSETF----IHRIGRTCR-L-NNKGKC(110)SIFLN(8)-IQKNN(9)--NNIKPFALKNNNGLVIT(92)-
PF3D7_0521700  --VAARGIDIQN-LRYLII---MTLS---DNINTF----FHKIGRVGR-D-GKNSLC-----IVLSA(8)-WFHTC(19)-VYIKESDYIKTINDMLE(82)-
PF3D7_1202000  --IISRGLDIDN-VIFILN---YSPP---TSPNDY----IHRSGRTGR-G-KEKGIC(95)KKNYVA(8)-EVIKN(15)-IITTIIGDVAKCDEGFI(90)-
PF3D7_0405000  --NISRGLDTVN-VNYVIN---FDMP---KHYNVL----THRIGRLAR-YNSRRGTV-----YHFIK(6)-MNKSGKQ---RNVNLIEQKRFKKNTLI(42)-
PF3D7_0721300  --IISRGIHFDS-LSVVIQ---YDPP---QILEEY----IHKVGRTAR-L-NKQGSA(39)-PNFLK(8)-FLHNHMQT--IVKSNNTLMEKGTSAFL(78)-
PF3D7_0504200  --IIYRGIDISN-ISHLFH---FDTP---TNIVVY----THRNGRLAR-G-ANTGHVY-------------IFKHFED---LVTRKIYELHKNK----(27)-
PF3D7_1307300  --ISSRGLNYPD-ITLIIQ---VNTP---ISREQY----IHRVGRTAR-S-NKKGMA-----IILLN(5)-FYQEIKD---LNIQKLNAQNYTLKNTN(90)-
PF3D7_1445200  --VLSRGIDLDK-CDLIIQ---LNCP---ISDITF----VHRSGRTAR-N-FKKGKC-----ICFIT(6)-WKTSL(7)--QDLQELDYLKSINEEDY(87)-
PF3D7_0321600  --LMSRGIDLIN-LNFVIN---YDCP---SDIFIY----IHRIGRCSS-V-NSEGQA-----ITFIL(6)-AFLIYTH---LKNKKRKIDKELEDFIL(79)-
PF3D7_1418900  --IACRGLDFSS-IDWVIH---FDFP---DNIETF----IHRSGRTGR-F-TNMGNS(170)ENYMC(8)-KDISH(28)-VEIKDDTPIMLPTKKRK(169)
PF3D7_1021500  --ILSRGIDIKG-IETVIN---YDVC---YDKYNY----MHRIGRACR-SDRKEGKA-----ITFFTS---ENIKYMK---EIIKFVKSSGTNIPSYL(67)-
PF3D7_1332700  --IISRGIDIPK-ISFVIN---FDFP---NDTVQY----IHRVGRTAR-A-NRKGLA-----ISFID(5)-SFNQVKN---IMKDKLKPYTLNKKEVL(38)-
PF3D7_0630900  --VAARGLDIPN-VNYIIQ---YDPP---DDSKEY----IHRVGRTCR-GQDSNGSA-----IIFLM(7)-NYLKFYN---IPINQFAYDPNKLINIQ(73)-
PF3D7_0218400  --VGARGLDLQD-IKIVIN---FDIC----SCKEY----IHRVGRTAR-A-GRSGKS-----ITFVT(8)-AIEKQLN---KKIDKFTDLDENDVLLY(31)-
PF3D7_0504400  --IASRGLDISN-VDLVIQ---CFPP---NYSAVY----IHRAGRTGR-A-NKKGTS(94)-RSLIN(8)-NFINK(20)-LNVNTFNKILQIKIDNK(74)-
PF3D7_1445900  --VASRGLDIKN-VKYVIN---FDFP---NQIEDY----VHRIGRTGR-A-GSHGAS-----FTFLTA---DKYRLAK---DLVKILRESEQPVPPQL(37)-
PF3D7_0527900  --VASKGLDFPS-IEHVIN---YDMP---KDIENY----VHRIGRTGR-C-GKTGIA-----TTFIN(8)-----------LDLKALLIEAKQKIPPF(67)-
PF3D7_1251500  --LASRGIDIDH-VLYVIN---YNVP---SNVIKY----VHRIGRTAR-I-GKEGIA(76)MITFKD(8)-TWFLT(20)-NNIKMNDHYENDGYDDV(87)-
PF3D7_1459000  --LLSRGIDVPS-ISLVIN---FDLP(19)NMETY----IHRIGRTGR-F-GTKGMA-----INFISK----------------NQMSHIKQIEEYYK(22)-
PF3D7_0209800  --LFGRGIDIER-VNIVIN---YDMP---ENSDSY----LHRVGRAGR-F-GTKGLA-----VTFVS(6)-ALNEV----------------------(23)-
PF3D7_0320800  --LFTRGIDIQS-VNVVIN---FDFP---KNSETY----LHRIGRSGR-Y-GHLGLA-----INLIT(6)-LYKIEVE---LGTEIQPIPNEIDPSLYT----
PF3D7_0422700  --IWGRGLDVQE-VSLVVN---YDLP---NSRESY----IHRIGRSGR-F-GRKGVA-----INFVK(5)-ILRDIEQ---YYSTQIDEMPMNITELL-----
PF3D7_1468700  --LLARGIDVQQ-VSLVIN---YDLP---ASPDTY----IHRIGRSGR-F-GRKGVA-----INFVT(7)-KLKKIES---YYSTQIEEMPLEVADYL-----
Consensus/80%   ..lhspGlsb.s.lphllp...bshs....p...a....hpR.GRstR...sp.G.s......pb.........p.........p..................
```

Key for residue coloring in alignment and consensus sequence

Description	Residues	Marker	Bold	Italic
Negative	DE	.	b	i
Aliphatic	ILV	|		
Positive	WKR	+		
Tiny	AGS	t	b	
Aromatic	FHWY	a	b	
Charged	DEHKR	c	b	i
Small	ACDGNPSTV	s	b	
Polar	CDEHKNQRST	p	b	
Big	EFIKLMQRWY	b		
Hydrophobic	ACFGHIKLMRTVWY	h	b	
