# Supplementary material for: A bioinformatic survey of RNA-binding proteins in Plasmodium
Source: BMC Genomics. 2015 Nov 2;16:890. doi: 10.1186/s12864-015-2092-1 (PMC4630921; doi:10.1186/s12864-015-2092-1)
Supplement: Additional file 7: — A structural and sequence-based comparison of PfPuf1 and PfPuf2. (a) A predicted model of PfPuf1 (using PDBID: 4dzs as a template) superimposed on a predicted model of PfPuf2 (using PDB ID: 3 k49 a template) at 3.4 root mean square deviation confirming the signature concave structure common to PUF domains. (b) A multiple sequence alignment of predicted PUF-domain sequences. PfPuf1 and PfPuf2 are 25 % identical at the domain level. (PDF 233 kb) [file 12864_2015_2092_MOESM7_ESM.pdf]

Additional file 7

(a)

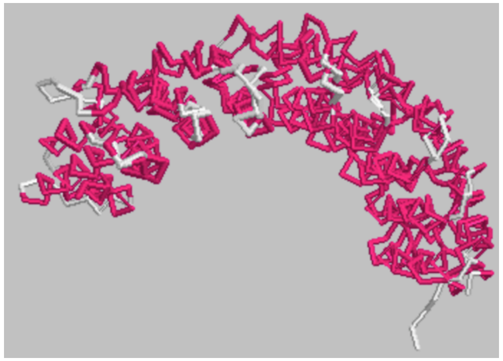

(b)

```
Puf1            NKRGV-QNKKNNYNKEN--YLSPISFTGNICKIAKDQTGCRILQRILEKKNPKHIEEIYNEALDHIIELMVDPFGNYLCQKLMEVCTSEQIEKIIDKSSD
Puf2            NKKDMFKNDEEKHNEEGQVHIDMSKVMYDIYFLCFHKNGCEYIIKKLKEDNKEEKEIILNSLLIDTMSLCPDIYGSYVAQSIFDLKDEKYKERFTDEFLK
Consensus/70%    NK+.h..N....aN.E...al....hhh.Ih.lh....GC.hl.+.L...N....E.IhN.hL..hh.Lh.D.aG.YlhQ.lh.l......E+hhD....

Puf1            QLINASISVHGTRTVQKLIEMIKTPSQIKKTTKALKNSIITLIKDINGNHVVQKCLITLTSHQCDFIYEAILNNCVEVSTHRHGCCVIQRCIDSANEAQK
Puf2            HTSFLTLHTYGCRLIQKSLESLSDEYKC-KIFKELQEDLIKYICHQNGNHVIQKCVEVLPCSYIDIIINIIEEYLPFLSSHAYGCRIVQRIYEIGNEQQI
Consensus/70%    .h..h.l.haGhRhlQK.lE.l.....h.KhhK.L...lI.hI...NGNHVlQKCl.hL....hDhIh.hI...h..lS.H.aGC.llQRhh..hNE.Q.

Puf1            ELFIRNISNNALDLVQDAFGNYVVQYILNLGNEKVNLEIANKLLPNIEELAVQKFSSNVVEKCLIIGNNKCRKLIINEI---LKKDKDILKQIILDPFGN
Puf2            NRLNEQIIKK-IHLIKNRYGNYVIQKCFEHSDDNIRFIITDEIVNDIYKLSSHKYACNIIEKILLKKEYKYKKKIIKKIVNDISDGKDNIINICKDCYGN
Consensus/70%    ..h...I....l.Ll...aGNYVlQ.hh.h....l.h.Ih..ll..I..L...Ka..NllEKhLl....Kh+K.II..I...l...KD.l..Ih.D.aGN

Puf1            YVIQRALSVASEPELTKLVEGIKPYIKELRNISSGKRIAWKLAKKH-
Puf2            FMMQKLLTTCRKKERNFIIKTIIENLDKLKDETYGKYILRAINNLEK
Consensus/70%    ahhQ+hL.hh...E...ll.hI...l..L+....GK.Ih..l.....
```
