# Supplementary material for: A bioinformatic survey of RNA-binding proteins in Plasmodium
Source: BMC Genomics. 2015 Nov 2;16:890. doi: 10.1186/s12864-015-2092-1 (PMC4630921; doi:10.1186/s12864-015-2092-1)
Supplement: Additional file 8: — A unified gene list identified as common between human and P. falciparum DOZI interactomes. Gene IDs from P. falciparum, along with their predicted function and biological process are provided. (PDF 49 kb) [file 12864_2015_2092_MOESM8_ESM.pdf]

Additional file 8

Gene ID	Functions	Cellular process
PF3D7_0217800	40S ribosomal protein S26 (RPS26)	Translation
PF3D7_0315100	Translation initiation factor 4E (eIF4E)	Translation
PF3D7_0320900	Histone H2A variant, putative (H2A.Z)	Chromatin assembly or disassembly
PF3D7_0626800	Pyruvate kinase (PyrK)	Glycolytic process
PF3D7_0810600	RNA helicase, putative	RNA binding
PF3D7_0814000	60S ribosomal protein L13-2, putative	Translation
PF3D7_0818200	14-3-3 protein (14-3-3I)	Histone binding
PF3D7_0822600	Protein transport protein SEC23 (SEC23)	Protein transport
PF3D7_0903700	Alpha tubulin 1	Structural constituent of cytoskeleton
PF3D7_0917900	Heat shock protein 70 (HSP70-2)	ATP binding
PF3D7_0923900	RNA-binding protein, putative	RNA binding
PF3D7_1008700	Tubulin beta chain	Structural constituent of cytoskeleton
PF3D7_1012400	Hypoxanthine-guanine phosphoribosyltransferase (HGPRT)	Purine ribonucleoside salvage
PF3D7_1015600	Heat shock protein 60 (HSP60)	Protein folding
PF3D7_1015900	Enolase (ENO)	Glycolytic pathway
PF3D7_1033600	myb2 transcription factor, putative (Myb2)	Transcriptional regulation
PF3D7_1105400	40S ribosomal protein S4, putative	Translation
PF3D7_1117700	GTP-binding nuclear protein RAN/TC4 (RAN)	Signal transduction
PF3D7_1120100	Phosphoglycerate mutase, putative (PGM1)	Glycolytic process
PF3D7_1128300	6-phosphofructokinase (PFK11)	Glycolytic process
PF3D7_1130200	60S ribosomal protein P0 (PfP0)	Translation
PF3D7_1134000	Heat shock protein 70 (HSP70-3)	Protein folding
PF3D7_1211800	Polyubiquitin (PfpUB)	Protein catabolism
PF3D7_1222300	Endoplasmin, putative (GRP94)	Cytoplasm
PF3D7_1224300	Polyadenylate-binding protein, putative (PABP)	RNA processing
PF3D7_1246200	Actin I (ACT1)	Structural constituent of cytoskeleton
PF3D7_1338300	Elongation factor 1-gamma, putative	Translation
PF3D7_1414300	60S ribosomal protein L10, putative	Translation
PF3D7_1424100	60S ribosomal protein L5, putative	Translation
PF3D7_1439900	Triosephosphate isomerase (TIM)	Glycolytic process
PF3D7_1451100	Elongation factor 2	Translation
PF3D7_1462800	Glyceraldehyde-3-phosphate dehydrogenase (GAPDH)	Glycolytic process
PF3D7_1465900	40S ribosomal protein S3	Translation
PF3D7_1468700	Eukaryotic initiation factor 4A (eIF4A)	Translation
PF3D7_1474900	Trailer hitch homolog, putative (CITH)	P-bodies
