# Supplementary material for: A bioinformatic survey of RNA-binding proteins in Plasmodium
Source: BMC Genomics. 2015 Nov 2;16:890. doi: 10.1186/s12864-015-2092-1 (PMC4630921; doi:10.1186/s12864-015-2092-1)
Supplement: Additional file 9: — A list of Caf1-CCR4-NOT complex genes identified by searching against human PPI database. The interactions were further used to perform BLASTp-search against PlasmoDB at E-value <0.1. (PDF 41 kb) [file 12864_2015_2092_MOESM9_ESM.pdf]

Additional file 9

Gene Names	PlasmoDB IDs	Human homolog Uniprot ID	Name of human homolog	Human homolog functional description	Number of interactions found in human	Number of human genes that have hits in Pf at E-value 0.1
BRF1	PF3D7_1449300	Q92994	BRF1	Transcription factor IIIB 90 kDa subunit	38	25
NOT1	PF3D7_1103800	A5YKK6	CNOT1	CCR4-NOT transcription complex subunit 1	136	98
CCR4	PF3D7_0519500	Q9ULM6	HCCR4	CCR4-NOT transcription complex subunit 6	182	146
CAF1	PF3D7_0811300	Q9UIV1	CAF1	CCR4-NOT transcription complex subunit 7	37	21
NOT5	PF3D7_1006100	O75175	CNOT3	CCR4-NOT transcription complex subunit 3	104	76
NOT2	PF3D7_1128600	Q9NZN8	CNOT2	CCR4-NOT transcription complex subunit 2	24	16
NOT4	PF3D7_1235300	O95628	CNOT4	CCR4-NOT transcription complex subunit 4	102	77
CAF16	PF3D7_1434000	Q8WWZ4	ABCA10		32	25
CAF40	PF3D7_0507600	Q92600	RQCD1	Cell differentiation protein RCD1 homolog	114	92
DCP1	PF3D7_1032100	Q9NPI6	DCP1A	mRNA-decapping enzyme 1A	30	22
DCP2	PF3D7_1308900	Q8IU60	DCP2	m7GpppN-mRNA hydrolase	21	13
PARN	PF3D7_1443500	O95453	PARN	poly(A)-specific ribonuclease	19	13
PABP	PF3D7_1224300	P11940	PAPB	Polyadenylate-binding protein 1	251	150
NOTx	PF3D7_1417200					
